# Artificial intelligence in nursing: a systematic review of attitudes, literacy, readiness, and adoption intentions among nursing students and practicing nurses

**DOI:** 10.3389/fdgth.2025.1666005

**Published:** 2025-09-25

**Authors:** Rabie Adel El Arab, Alya H. Alshakihs, Sarah H. Alabdulwahab, Yasmeen S. Almubarak, Shahad S. Alkhalifah, Amany Abdrbo, Salwa Hassanein, Mette Sagbakken

**Affiliations:** ^1^ Almoosa College of Health Sciences, Al Ahsa, Saudi Arabia; ^2^ Faculty of Health Sciences, Department of Nursing and Health Promotion, Oslo Metropolitan University, Oslo, Norway

**Keywords:** artificial intelligence, nursing, attitudes, AI literacy, AI readiness, adoption intentions, technology acceptance, nursing students

## Abstract

**Background:**

Artificial intelligence (AI) could reshape healthcare delivery, but its adoption depends on nurses' attitudes, literacy, readiness, and intentions.

**Methods:**

Following PRISMA 2020, we searched six databases from inception to May 2025 and undertook thematic synthesis. A non-systematic horizon scan (June–August 2025) informed interpretation only.

**Results:**

Thirty-seven studies met inclusion: 28 analytical cross-sectional surveys, 8 qualitative studies, and 1 quasi-experimental trial.

Nursing students generally held moderately positive attitudes towards AI; senior students were more enthusiastic than juniors, and men more than women. Students reported moderate literacy and readiness; prior AI training and stronger computer skills correlated with more favourable attitudes and greater adoption intentions, whereas anxiety dampened readiness. Many students doubted AI's ability to outperform humans in routine tasks and flagged integrity risks, underscoring the need for age-appropriate instruction and safeguards. Practising nurses expressed moderate safety and error concerns but showed greater optimism among younger staff; across studies, nurses consistently argued AI should augment—not replace—human empathy and judgement. Targeted training substantially improved, and largely maintained, AI knowledge; leadership endorsement and phased, user-centred roll-outs strengthened readiness, while outdated infrastructure, resource constraints, ethical/privacy concerns, and fear of deskilling impeded progress. Determinants of attitudes and intentions clustered around perceived usefulness/performance and effort expectancy, self-efficacy, digital literacy, and facilitating conditions. The horizon scan added signals of a preparedness–impact gap among nurse leaders, syllabus/policy language as a faculty readiness multiplier, role-specific adoption gaps (e.g., lower use among head nurses despite positive attitudes), and coexistence of high AI anxiety with positive attitudes in students.

**Conclusion:**

Global nursing exhibits guarded optimism grounded in moderate literacy and readiness yet constrained by infrastructural, ethical, and pedagogical barriers. Adoption is driven by perceived usefulness, self-efficacy, and enabling environments, with anxiety and demographics moderating engagement. Priorities include embedding longitudinal AI competencies in curricula, iterative hands-on training, robust governance/ethics, and modernised infrastructure. Evidence dominated by cross-sectional designs and a narrow set of countries should be strengthened through longitudinal and experimental studies that validate psychometrics cross-culturally and link self-reports to objective use and patient-safety outcomes.

## 1 Introduction

Artificial intelligence (AI) is rapidly emerging as a transformative force in healthcare, with broad applications from diagnostics and predictive analytics to patient care management (1–7). Globally, the adoption of AI in healthcare is uneven, with high-income countries often spearheading implementation of AI solutions, while many low- and middle-income countries (LMICs) face infrastructure and resource barriers that can hinder progress (8, 9). This disparity raises the risk of a digital divide: without concerted efforts, AI could exacerbate existing gaps in healthcare quality between well-resourced and resource-constrained settings.

**Table 1:** SPIDER framework for study eligibility.

Component	Definition
S (Sample)	Undergraduate and postgraduate nursing students; practising nurses (staff nurses, nurse leaders, nurse educators)
PI (Phenomenon of Interest)	Attitudes, perceptions, AI literacy, readiness, and behavioural intentions regarding the adoption of artificial intelligence in clinical practice and nursing education
D (Design)	Analytical cross-sectional surveys; quasi-experimental trials; qualitative studies (individual interviews, focus groups); mixed-methods research.
E (Evaluation)	Quantitative measures (validated scales such as GAAIS, AILS, MAIRS-MS, AIAS, UTAUT/UTAUT2 subscales); thematic findings on determinants, barriers, and facilitators of AI adoption
R (Research type)	Primary empirical studies published in peer-reviewed journals from inception to May 2025, and in English

**Table 2:** Inclusion and exclusion criteria.

Inclusion criteria
Publication type: Primary empirical studies (analytical cross-sectional surveys; quasi-experimental trials; qualitative studies; mixed methods)
Focus: Investigation of one or more of the following in relation to artificial intelligence in clinical or educational nursing contexts: attitudes, perceptions, AI literacy, readiness, or behavioural intentions
Population: Undergraduate or graduate nursing students; practising nurses (staff nurses, nurse leaders, educators)
Context: Clinical practice or formal nursing education settings
Language: English
Time frame: from inception to 30 May 2025
Exclusion criteria
Publication type: Reviews, meta-analyses, commentaries, editorials, protocols, conference abstracts, dissertations, gray literature
Focus: Studies solely describing technical performance of AI algorithms without reporting nursing stakeholders' attitudes, literacy, readiness, or intentions
Population: Non-nursing health professionals or students; mixed-profession samples without disaggregated nursing data
Language: Publications in languages other than English

In nursing practice, AI-driven technologies—including clinical decision support systems, continuous monitoring tools, and workflow automation—promise to enhance decision-making, improve efficiency, and ultimately elevate patient safety and outcomes (10–13). Nurses comprise the largest segment of the healthcare workforce and are on the front lines of patient care (14, 15); therefore, their readiness to understand and use AI will critically determine how successfully these innovations translate into real-world improvements.

At the level of individual practitioners and students, attitudes, perceptions, and literacy regarding AI are emerging as critical determinants of adoption. Early evidence suggests that nurses generally have a cautiously positive view of AI's potential, yet significant knowledge gaps and uncertainties persist (11). Nurses' perceptions of AI appear to vary widely depending on their exposure and experience (16, 17). Indeed, several key barriers to AI acceptance in healthcare have been documented, including ambiguity in regulations and legal liability, lack of organizational support or training, data privacy and security concerns, and the fear that AI could depersonalize care or make flawed decisions without human oversight (12, 18–20). In one review, regulatory uncertainties, liability risks, and organizational resistance were identified as significant impediments to healthcare providers' uptake of AI, alongside worries that AI might replace human judgment in critical scenarios or fail to address the empathetic aspects of nursing care (21). These barriers not only hinder the implementation of AI technologies but can also negatively impact healthcare providers' morale, job satisfaction, and willingness to engage with such innovations.

To better explain and predict technology acceptance in such contexts, researchers often draw on established theoretical models. The Technology Acceptance Model (TAM) offers a robust, validated framework for understanding how users come to accept and use new technologies (22). In TAM, two beliefs—perceived usefulness (the extent to which the technology is seen to improve job performance) and perceived ease of use (the degree to which the technology is seen as free of effort)—are posited as key drivers of an individual's attitude toward using the technology, which in turn influences their behavioral intention to use it (22). Unified Theory of Acceptance and Use of Technology (UTAUT) is another influential model, integrating eight earlier technology acceptance theories to explain up to about 70% of the variance in usage intentions. UTAUT identifies performance expectancy (similar to perceived usefulness), effort expectancy (similar to ease of use), social influence (the effect of peers and superiors), and facilitating conditions (the availability of organizational and technical support) as core determinants of technology adoption behaviors (23). Both TAM and UTAUT highlight that beyond the technical merits of AI, human factors—such as a nurse's belief in AI's value, their comfort with using it, and the support they feel around them—are pivotal in determining adoption.

Despite burgeoning interest and isolated studies on this topic, there remain important gaps in the evidence regarding nurses' and nursing students' acceptance of AI. The literature to date is fragmented by geography and subgroup, often limited to single-country studies or focused on either students or practicing clinicians in isolation. In light of the rapid advancement of AI in health care and the critical role of nurses in its uptake, a systematic synthesis of the available evidence is necessary. Such a synthesis can identify common themes and disparities across different countries and contexts and pinpoint where knowledge gaps persist. Ultimately, understanding nurses' attitudes, literacy, and readiness for AI on a global level will inform targeted strategies in education, training, and policy to support the safe and effective integration of AI in nursing.

## 2 Aim

This systematic review will synthesise global quantitative and qualitative evidence on the attitudes, perceptions, AI literacy, readiness, and behavioural intentions of nursing students and practicing nurses toward artificial intelligence in both clinical and educational contexts; identify key determinants, barriers, and facilitators influencing AI adoption; and propose evidence-based curricular, professional development, and policy strategies for the safe, ethical, and effective integration of AI into nursing education and practice.

## 3 Objectives

To assess nursing students' and nurses' attitudes toward AI in clinical and educational contexts.

To evaluate AI Literacy, Readiness & Intention of nursing students and practicing nurses to adopt AI technologies

To identify Determinants, Barriers & Facilitator factors influencing AI adoption.

## 4 Methods

### 4.1 Study design

This systematic review was conducted in accordance with the Preferred Reporting Items for Systematic Reviews and Meta-Analyses (PRISMA) 2020 statement to ensure rigor and transparency (24). Our approach followed these sequential steps: eligibility criteria formulation, systematic literature search, study selection, data extraction, methodological quality and risk-of-bias appraisal, and we applied thematic synthesis (25).

### 4.2 The sample, phenomenon of interest, design, evaluation, research type framework

We defined our eligibility criteria using the Sample, Phenomenon of Interest, Design, Evaluation, Research type tool (SPIDER) (26). Unlike Population, Intervention, Comparison, Outcome (PICO), SPIDER accommodates the conceptual breadth of studies exploring attitudes, literacy, readiness, and behavioural intentions toward AI, ensuring we capture both numerical measures (e.g., survey scores) and rich, contextual insights (e.g., interview themes).

Applying SPIDER allowed us to include a wide spectrum of primary empiric work from large cross-sectional surveys to in-depth qualitative inquiries conducted from inception to May 2025. This framework ensured systematic identification of studies that directly address how nursing students and practising nurses perceive, understand, and intend to use AI in clinical and educational settings, while also illuminating key determinants, barriers, and facilitators of adoption (see Table 1).

### 4.3 Inclusion and exclusion criteria

To ensure we captured only directly relevant, high-quality primary studies on nursing students' and practising nurses' engagement with AI, we applied the following criteria (see Table 2):

### 4.4 Information sources and search strategy

We searched PubMed/MEDLINE, Embase, CINAHL, Scopus, Web of Science, and IEEE Xplore, to capture peer-reviewed and emerging preprint literature in biomedical, nursing, and technology domains. Our strategy combined controlled vocabulary (MeSH/Emtree terms for “Artificial Intelligence”, “Machine Learning”, “ChatGPT” etc.) with keywords for nursing roles (“nurs*”, “nursing student*”) and acceptance constructs (“attitude*”, “readiness”, “UTAUT2”, “TAM”). Complete search strings are provided in Supplementary Appendix S1.

### 4.5 Study selection

**Figure 1:**
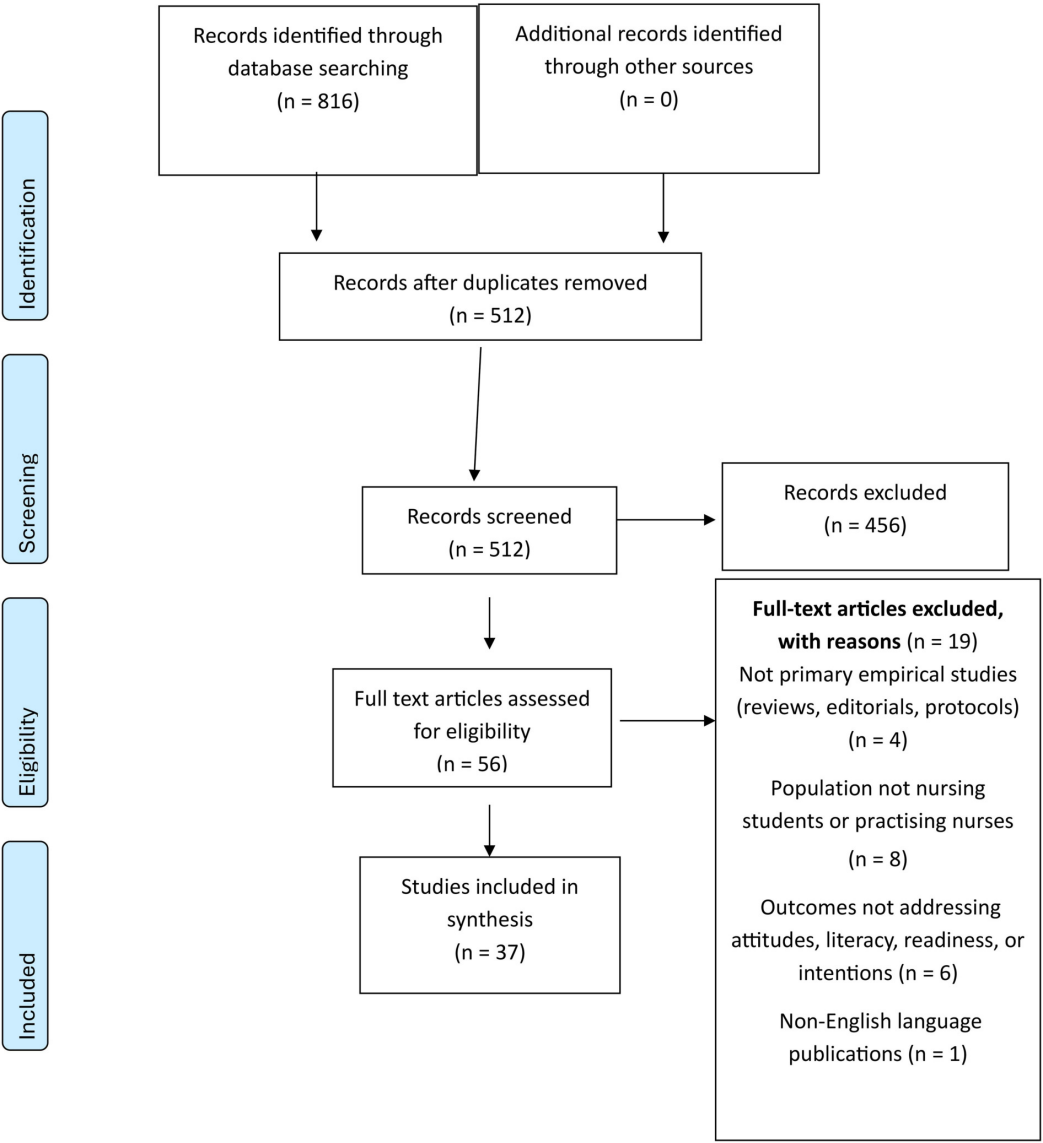
PRISMA 2020 flow diagram of study identification.

All records were uploaded into Rayyan for de-duplication (27). Two reviewers independently screened titles and abstracts (RAE and AHA), then full texts, against SPIDER-based eligibility criteria; inter-rater agreement was excellent (Cohen's κ > 0.80). Conflicts were resolved through consensus or, if needed, adjudication by a third reviewer (SSA or SHA). The detailed study-selection process is illustrated in Figure 1.

### 4.6 Data extraction and synthesis

We used a piloted Excel form to capture author, year, country; study design and setting; sample characteristics; data collection period; theoretical framework; measurement tools; key quantitative outcomes; and qualitative themes. Dual independent extraction ensured data integrity, with discrepancies reconciled by discussion. Quantitative findings were tabulated and summarized descriptively. Given the diversity of measurement instruments, differing in item count, response formats, and underlying constructs we transformed each scale's meaning and standard deviation (SD) into standardized z-scores before any pooled descriptive summaries. We also conducted subgroup syntheses by instrument family (e.g., General Attitudes Towards AI Scale (GAAIS) variants, AILS variants, UTAUT-based tools) and reported findings separately where psychometric properties or cultural adaptations differ substantially. Finally, we assessed each tool's cross-cultural validity by noting whether local translations underwent forward–back translation and confirmatory factor analysis; where such validation was absent, we downgraded confidence in those estimates.

Qualitative data were imported into Atlas.ti (28) and subjected to thematic synthesis following Thomas & Harden's three-stage approach (25) open coding, development of descriptive themes, and generation of higher-order analytical themes to integrate diverse evidence into an actionable conceptual framework. To ensure balanced integration of quantitative and qualitative evidence, we employed a convergent integrated approach: quantitative effect sizes and proportions were first mapped onto our thematic framework, then qualitative themes were used to contextualize and explain these numerical findings. Each theme's strength was determined by the number of studies and range of effect estimates (for quantitative data) alongside depth of illustrative quotes and thematic saturation (for qualitative data), allowing us to weigh and triangulate evidence across methodologies.

### 4.7 Quality assessment and risk of bias

In order to ensure a rigorous and transparent evaluation of study quality, we employed the Joanna Briggs Institute (JBI) Critical Appraisal tools tailored to each study design, as described in the JBI Manual for Evidence Synthesis (29–33). For the analytical cross-sectional surveys, we applied the eight-item JBI Analytical Cross-Sectional Checklist, scoring each item dichotomously (“Yes”/“No”) to yield a total from 0 to 8. The single quasi-experimental trial was appraised using the nine-item JBI Quasi-Experimental Studies Checklist, which adds criteria for causal linkage, control group presence, multiple measurement points, consistency of outcome measurement, and instrument reliability. For the qualitative inquiries, we used the ten-item JBI Critical Appraisal Checklist for qualitative research. Two reviewers (RAE, YSA) independently conducted all appraisals, discrepancies were resolved by consensus or, if needed, adjudication by a third reviewer (AA or SH). To characterize internal validity, we assessed risk of bias separately from methodological quality. For cross-sectional and quasi-experimental designs, we applied the JBI bias domains for observational studies and the ROBINS-I tool for non-randomized interventions (34). Qualitative studies were judged against five JBI bias domains; selection, investigator, data-collection, analysis, and reporting bias with overall risk of bias synthesized accordingly. Extracted item-level responses, domain judgments, and overall risk ratings were recorded in a pre-piloted data collection form, and summary statistics were generated to convey the distribution of quality and bias ratings across the body of evidence. This structured, multi-tool approach provides a robust foundation for interpreting the strength and trustworthiness of our subsequent syntheses.

### 4.8 Post-search surveillance

During peer review we performed a non-protocol, nonsystematic top-up check (June–August 2025) and narratively summarized relevant studies in the Discussion; these were not included in screening counts, tables, or quantitative syntheses.

## 5 Results

A total of 37 studies (35–71) (combined *N* ≈ 10290) published between 2022 and May 2025 met our inclusion criteria. Eighteen studies (*n* = 159–1,713 per study) sampled undergraduate nursing students (35, 40, 42, 43, 49, 50, 53, 57, 62–71), and Eighteen studies (*n* = 20–600) surveyed practicing nurses or nurse leaders (36, 38, 39, 41, 44–48, 51, 52, 54–56, 58–61). One study (37) included both nurses and nursing students (counted only once).

Of the 37 studies, 28 (75.7%) were analytical cross-sectional surveys, 1 (2.7%) was a quasi-experimental trial, and 8 (21.6%) were qualitative studies.

Geographic distribution was heavily weighted towards Turkey, Egypt, and Saudi Arabia, with additional contributions from Republic of Korea, Jordan, Bangladesh, Palestine, Croatia, Philippines, Australia, Greece, China, Bahrain, Iraq, Kuwait, Lebanon, Oman and United Arab Emirates. Notably, there are no studies from the Americas (North, Central or South America), western Europe, and none from sub-Saharan Africa, all other regions are unrepresented. This geographic skew indicates that cultural, infrastructural, and educational contexts in under-represented regions particularly the Americas and sub-Saharan Africa may produce differing attitudes, literacy levels, and readiness for AI among nurses; therefore, our findings primarily reflect Middle Eastern and North African perspectives and should not be generalized to the global nursing workforce. Instruments spanned over 30 scales, including the GAAIS, the Artificial Intelligence Literacy Scale (AILS), the Medical Artificial Intelligence Readiness Scale–Medical Students (MAIRS-MS), the Artificial Intelligence Attitude Scale (AIAS), UTAUT, and its extension, UTAUT 2, with Cronbach's α ranging from 0.766 to 0.986. Notably, however, one Ten-Item Personality Inventory (TIPI) subscale in Tsiara et al. (42) exhibited an α of 0.016 far below the acceptable threshold indicating essentially no internal consistency. We therefore excluded TIPI-derived personality correlations from our quantitative synthesis and interpret any related findings with extreme caution. (see Supplementary Appendix S2: Characteristics of Included Studies).

## 6 Thematic synthesis

This thematic synthesis integrates quantitative and qualitative findings from studies exploring nursing students' and practicing nurses' attitudes, perceptions, AI literacy, readiness, behavioural intentions, and the determinants influencing AI adoption. Four major themes emerged: (1) Attitudes and Perceptions, (2) AI Literacy, Readiness, and Behavioural Intentions, (3) Determinants, Barriers, and Facilitators, and (4) Lived Experiences and Ethical Reflections. Each theme and subtheme synthesize evidence to illuminate how nurses perceive AI, their readiness to integrate it, and factors that facilitate or hinder its adoption, ultimately guiding educational, clinical, and policy strategies for effective AI integration in nursing.

### 6.1 Theme 1: attitudes and perceptions

Attitudes and perceptions serve as the cognitive and affective lens through which nursing students and nurses evaluate AI's promise and pitfalls. By dissecting these sentiments, educators and policymakers can tailor interventions that bolster trust in AI's benefits while addressing ethical, practical, and emotional reservations.

#### 6.1.1 Nursing Students' attitudes

Undergraduate nursing students consistently report moderately positive attitudes toward AI, with item-mean scores ranging from 3.03 ± 0.53 to 3.86 ± 0.62 on 1–5 scales (49, 69) and a sum-score mean of 64.5 ± 11.7 on the 20–100 GAAIS scale (70). Demographic gradients further nuance this picture. Male students consistently outscore female peers by approximately 0.6–0.8 points on positive-attitude subscales for instance, 4.16 ± 0.69 vs. 3.59 ± 0.57 at Yeditepe University (*p* < 0.001) suggesting sociodemographic influences on AI engagement (63). Likewise, academic seniority correlates with more favorable attitudes: fourth-year students demonstrate higher GAAIS and readiness scores than lower years (*F* = 3.75; *p* = 0.011), and third- and fourth-year cohorts outperform first- and second years on both AI literacy and positive-attitude measures (69). At the psychosocial level, performance expectancy (belief that AI will enhance job performance) and self-efficacy (confidence in using AI tools) emerge as the strongest predictors of a positive attitude, together explaining nearly half of its variance (β ≈ 0.37 and β ≈ 0.28, respectively; *R*^2^ ≈ .48) in Korean nursing students (68). This underscores the importance of designing curricula and hands-on experiences that both demonstrate AI's concrete benefits and build students' confidence in its use.

#### 6.1.2 Practicing Nurses' attitudes

Practice nurses exhibit a cautiously optimistic stance toward AI: a targeted educational intervention elevated the proportion of nurses with “satisfactory” AI knowledge from 16.3% at baseline to 82.8% immediately post-workshop (*χ*^2^ = 125.0; *p* < 0.001) and lifted positive-attitude prevalence from 26.1% to 81.8% (*F* = 65.2; *p* < 0.001), yet without reinforcement, attitudes waned to 63.5% at three months demonstrating that while intensive training can powerfully shift beliefs, periodic “booster” sessions are essential for enduring attitude gains (56).

Demographically, attitude heterogeneity is marked. In northern Saudi Arabia, registered nurses' mean attitude-item ratings spanned 2.87–3.66; female nurses consistently reported lower positivity than males (*χ*^2^ = 4.67; *p* = 0.03), whereas younger practitioners were significantly more receptive than their older peers (*χ*^2^ = 9.31; *p* = 0.02), underscoring the need for demographically tailored communication and support strategies (59). At the heart of these attitudes lies perceived clinical utility: Egyptian staff nurses rated “AI reduces medical errors” at 4.04 ± 0.96 on a 5-point scale, and this perception correlated very strongly with overall attitude (*r* = 0.715; *p* < 0.001), indicating that tangible examples of AI's patient-safety benefits can meaningfully elevate nurse buy-in (55). Complementing this, 85% of Saudi nurses in focus groups linked AI directly to improved diagnostic accuracy and workflow efficiency, a consensus that forms the cornerstone of their positive outlook (60).

Ethical and organizational dimensions further refine these perspectives. Among 415 Egyptian bedside nurses, higher ethical-awareness scores not only predicted greater innovative work behaviors (*B* = 2.567; *p* = 0.013) but also amplified the effect of positive attitudes on innovation (interaction *B* = 0.038; *p* = 0.002), demonstrating that ethics training multiplies the practical impact of favorable attitudes (48).

Moreover, 70% of Saudi participants highlighted strong leadership commitment visible investment, clear communication, and AI “champions” as a critical enabler, with those in well-supported units reporting markedly higher attitude-item means (60). Yet, concerns about privacy and governance temper enthusiasm: Saudi nurses rated “AI is used to spy on people” at 2.88/5 and “organizations use AI unethically” at 2.87/5, and higher worry scores were significantly associated with lower overall attitudes particularly among female staff pointing to the imperative for transparent data-protection policies and ethics governance to sustain nurse confidence (59).

### 6.2 Theme 2: AI literacy, readiness, and behavioural intentions

Competence (literacy), confidence (readiness), and motivation (intentions) jointly shape whether and how nursing students and professionals adopt AI tools.

#### 6.2.1 AI literacy among nursing students

AI literacy among undergraduate nursing students has been measured most rigorously in two large cross-sectional surveys. Akca Sumengen et al. (63) assessed 205 Yeditepe University students using the 12-item Artificial Intelligence Literacy Scale (AILS), finding a mean total score of 58.68 ± 8.12. Those with prior AI coursework scored significantly higher (62.67 ± 8.46) than their untrained peers (57.32 ± 7.55; *p* < 0.001), and regular AI users outperformed non-users (60.52 ± 7.87 vs. 55.55 ± 7.59; *p* < 0.001). Third-year students also demonstrated greater usage proficiency (14.84 vs. 13.46; *p* = 0.023) and more positive attitudes on the General Attitudes Towards AI Scale (GAAIS; 3.84 vs. 3.53; *p* = 0.036) than first years. Cronbach's α for AILS in this cohort exceeded 0.93, and literacy–attitude correlations ranged from *r* = 0.318 to 0.519 (all *p* < 0.001), attesting to both the instrument's reliability and the robust link between AI knowledge and positive disposition. El-Sayed et al. (64) surveyed 596 Egyptian students with a 31-item AI literacy measure and reported similarly strong internal consistency (α = 0.93) and a substantial correlation with career self-efficacy (*r* = 0.568; *p* < 0.01). Across these studies, literacy–attitude correlations consistently cluster around *r* ≈ 0.40–0.52, providing a reproducible benchmark for the relationship between competence and favorable AI perceptions.

#### 6.2.2 AI literacy among practicing nurses

In clinical settings, perioperative and hospital-based nurses exhibit moderate AI literacy that nonetheless correlates meaningfully with attitudes and self-reported practice readiness. Kahraman et al. (52) administered AILS to 505 Turkish perioperative nurses and found a mean total score of 44.35 ± 5.88. Subscale scores for awareness, use, evaluation, and ethics ranged from 10.22 to 11.94, and reliability was solid (α = 0.821). Male nurses and those with prior AI exposure scored significantly higher (*p* < 0.05), underscoring the driving role of familiarity and experience. Mariano et al. (37) surveyed 349 Saudi nurses, faculty, and students, reporting an average AI knowledge test score of 7.37 ± 2.67 out of 10; this knowledge correlated positively with attitudes (*r* = 0.451; *p* < 0.01) and practice behaviors (*r* = 0.404; *p* < 0.01). Meanwhile, Abou Hashish and Alnajjar (57) found moderate digital health literacy and AI attitudes = 3.42 ± 0.54 among 266 Saudi nursing students, with significant correlations between digital transformation knowledge and AI attitudes (*r* = 0.354; *p* < 0.001). These findings highlight that even moderate-level AI competence in practicing nurses is strongly associated with positive outlooks and willingness to integrate AI into care.

#### 6.2.3 Readiness and behavioural intentions

Behavioral intention to adopt AI in nursing is driven by a blend of attitudes, facilitating conditions, and affective factors, as demonstrated by multiple path-analysis and regression models. In South Korea, Kwak, Seo, et al. (68) surveyed 210 students and found that positive attitude (*β* = 0.485; *p* = 0.009) and facilitating conditions (*β* = 0.117; *p* = 0.045) together explained 30% of the variance in intention (*R*^2^ = 0.30). Alenazi and Alhalal (43) applied UTAUT2 to 500 Saudi students, accounting for 34% of intention variance (*R*^2^ = 0.342) with hedonic motivation (*β* = 0.371) and habit (*β* = 0.458) as the strongest predictors and demonstrated that intention strongly drove actual AI use (*β* = 0.702; *p* < 0.001). Cho and Seo (49) revealed dual mediation in a sample of 180 Korean students: acceptance attitude exerted an indirect effect of 0.133 on intention, and anxiety influenced intention via attitude (indirect = 0.026; 95% CI excludes zero), reducing the direct perception on intention path from b = 0.478 to b = 0.280. Among practicing nurses, Oweidat et al. (41) showed that knowledge (*β* = 0.23), attitudes (*β* = 0.35), practices (*β* = 0.28), and barriers (*β* = –0.14) jointly explained 56% of variance in intent to stay (*R*^2^ = 0.56), underscoring that favorable AI engagement fosters workforce retention. Finally, Ünal and Avcı (38)found a strong inverse relationship between AI anxiety and readiness in neonatal nurses (Spearman *r* = –0.549; *p* < 0.01), with both scales demonstrating excellent reliability (α > 0.98). In a landmark multicountry survey, Al Omari et al. (50) analyzed responses from 1 713 Arab nursing students and reported perception (β = 0.295), attitude (β = 0.211), and knowledge (β = 0.061) as independent predictors of intention (*R*^2^ = 0.342), illustrating broad geographic consistency in the drivers of AI adoption.

Collectively, these results reveal that AI-literacy instruments boast high internal consistency (α > 0.80) and validated factor structures, while literacy–attitude correlations (*r* ≈ 0.40–0.52) and readiness–anxiety links (*r* = –0.549) are reproducible across contexts. Predictive models explain 30%–56% of variance in adoption intentions (and up to 70% of actual use), signaling that interventions targeting attitudes, enablers, and anxiety reduction can have substantial impact.

### 6.3 Theme 3: determinants, barriers, and facilitators

Successful AI adoption depends on individual, organizational, and cultural factors.

#### 6.3.1 Demographic & educational determinants

Among undergraduates, males consistently outperform females on both AI literacy and attitudes: at Yeditepe University (*n* = 205), literacy averaged 58.7 ± 8.1 and positive attitudes 3.66 ± 0.61, with men scoring higher on both (*p* < 0.001) (63). In other study, overall attitude was 66.2 ± 7.4, with females scoring ∼3 points above males (*B* =  + 2.95; *p* < 0.001) and each academic year adding +0.60 to attitude (*p* = 0.024) (62). Fourth-year Turkish students (MAIRS-MS 76.9 ± 13.6) outperformed second years (*F* = 3.75; *p* = 0.011), and readiness correlated with attitudes (*r* = 0.33; *p* < 0.01) (69). Personality also matters: in Greece (*n* = 159), openness linked to positive attitudes (*r* = 0.166; *p* = 0.043) and extraversion inversely to negative attitudes (*r* = –0.181; *p* = 0.030) (42), and in Turkey (*n* = 314) AI literacy correlated with both attitudes (*r* = 0.433; *p* < 0.01) and anxiety (*r* = 0.322; *p* < 0.01), with anxiety partially mediating literacy's effect on attitude (71).

Among practicing nurses, similar patterns emerge. In Turkiye's perioperative units (*n* = 505), total literacy was 44.4 ± 5.9; men scored higher on all subscales (*p* < 0.05) and age correlated inversely with literacy (*r* = –0.18 to −0.06; *p* < 0.05) (52). Among 107 Neonatal nurses, higher anxiety predicted lower readiness (*r* = –0.549; *p* < 0.01) (38). Among 187 nurse leaders, multiple regression analyses revealed that leadership role, academic qualifications, age group, and professional experience collectively explained 37.5% of the variance in AI readiness and 40% in perceived benefits. Specifically, holding a master's in nursing vs. bachelor's in nursing occupying nurse manager or educator roles, being over 35 years of age, and having over 15 years of experience were all significant positive predictors (47).

#### 6.3.2 Organizational & infrastructural factors

Across both academic and clinical settings, the successful adoption of AI hinges not only on individual readiness but also on the organizational and infrastructural ecosystems in which nursing students and practicing nurses operate. While nurses tend to emphasize system-level enablers rooted in workplace functionality, nursing students often frame AI integration through the lens of institutional preparedness and educational exposure.

Among practicing nurses, visible leadership commitment and organizational endorsement emerged as critical determinants of AI engagement. In a qualitative study, 70% of nurses underscored that active leadership support including managerial advocacy, infrastructure investment, and phased deployment plans was a decisive factor in shaping their willingness to engage with AI systems (60). This emphasis on top-down facilitation reflects nurses' proximity to frontline implementation realities, where trust in institutional backing is essential for overcoming initial resistance and building confidence in AI tools.

In contrast, nursing students framed organizational readiness primarily around educational infrastructure and formal AI training opportunities. In a large-scale survey conducted in the West Bank, 79% of Palestinian nursing students advocated for the integration of AI-focused modules into undergraduate curricula, citing a strong demand for hands-on, context-specific workshops to bridge the gap between theoretical knowledge and clinical applicability (53). Abou Hashish and Alnajjar (57) did not quantify training demand explicitly, nursing students in their study demonstrated high digital proficiency (digital skills mean = 4.09 ± 0.74) and digital health literacy (mean = 3.72 ± 0.76), suggesting that academic readiness for AI is present, but institutional offerings may lag behind student expectations.

Infrastructural limitations including outdated hardware, poor system interoperability, and limited access to AI-enabled tools represented a shared barrier for both groups, though expressed through different operational lenses. Practicing nurses in Ramadan et al. (60) identified infrastructural gaps as a significant barrier to safe implementation, particularly in settings where digital integration was uneven or underfunded. Similarly, more than half of the students in the Salama et al. (53) study expressed reservations about the technical reliability of AI systems, particularly regarding error rates in decision support and diagnostic accuracy.

These findings delineate a dual but complementary set of infrastructural priorities: nurses demand system-level reliability, strategic leadership, and clinical alignment, while students seek structured educational pipelines, experiential learning, and future-oriented curricular integration. Bridging these domains requires targeted investments not only in physical infrastructure but also in pedagogical reform and workforce-level leadership without which the promise of AI will.

#### 6.3.3 Ethical & cultural influences

##### 6.3.3.1 Nursing students

Although cultural comparisons have not been directly tested, quantitative studies illuminate how ethics-related constructs shape but do not solely determine undergraduate nurses' engagement with AI. In a sample of 314 Turkish nursing students, AI anxiety (α = 0.942) was found to partially mediate the positive relationship between AI literacy and general AI attitudes: higher literacy promoted more favorable attitudes in part by alleviating anxiety, rather than anxiety itself acting as an independent driver (indirect effect's 95% CI excludes 0) (71). In South Korea, 189 nursing undergraduates scored highly on the Test for AI Ethics Awareness (mean 3.27 ± 0.24), yet hierarchical regression revealed that only positive attitudes (*β* = 0.49; *p* < 0.001) and self-efficacy (*β* = 0.22; *p* = 0.002) significantly predicted intention to use AI indicating that ethics awareness exerts its influence primarily through attitudinal and efficacy pathways, rather than as a standalone predictor (35).

##### 6.3.3.2 Practicing nurses

Practicing neonatal nurses emphasized the urgent need for organizational structures that support culturally sensitive, context-aware AI integration. In a study in Saudi Arabia, participants highlighted the dependence of safe AI adoption on robust infrastructure, sustained training, and clear governance protocols. While ethical considerations were woven into discussions of professional autonomy and system readiness, nurses also noted that cultural expectations around family-centred care influence how AI tools are perceived and integrated, though explicit parental involvement in AI-driven decision-making was not formally structured (46). Jordanian nurses voiced consistent concerns regarding data privacy, security breaches, and the risk of job displacement in an evolving AI landscape. Findings from individual interviews and focus group discussions underscored the need for structured, organization-wide education and training initiatives to ensure the safe and effective integration of AI technologies into nursing practice, moving beyond sporadic sessions toward sustained professional development (58). In a cross-sectional study of 415 Egyptian bedside nurses, higher scores on the Ethical Awareness of AI Scale (mean 43.85 ± 3.39) significantly predicted greater innovative work behaviours (*B* = 2.567; *p* = 0.013). Moreover, ethical awareness moderates the relationship between general attitudes toward AI and innovation (interaction *B* = 0.038; *p* = 0.002), suggesting that ethical competencies may enhance the translation of positive AI attitudes into tangible clinical innovation (48).

### 6.4 Theme 4: lived experiences and ethical reflections

Qualitative narratives reveal why nurses hold their views, complementing quantitative metrics. In their semi-structured interviews with thirteen first- and second-year students, Summers et al. (65) (*n* = 13) discerned six discrete themes Educational Impact; Equity; Ethical Considerations; Technology Integration; Safety & Practical Utility; and Generational Differences through which participants articulated a nuanced appreciation for AI's capacity to produce rapid summaries and streamline academic tasks, while voicing deep concern that overreliance on generative tools could erode critical thinking skills and compromise academic integrity.

Among practicing nurses, multiple inquiries further enrich our understanding. Rony, Kayesh, et al. (61), (*n* = 23) identified ten thematic domains encompassing readiness, perceived benefits and concerns, ethical safeguards, and the need to maintain a human–AI balance. Ramadan et al.'s (60) (*n* = 48) distilled four themes understanding, facilitators, barriers, and attitudes and from these crafted an extended Technology Acceptance Model for Artificial Intelligence in Nursing (TAM-AIN) framework that integrates ethical alignment, organizational readiness, and professional-identity preservation. Almagharbeh et al. (58) (*n* = 25 plus three focus groups) surfaced three overarching themes: AI as an efficiency enabler, ethical and practical challenges, and the urgent need for structured training. In high-risk neonatal units, Alruwaili et al. (46) (*n* = 33) described five themes: AI serving as a complementary “second opinion”, the evolution of nurses' consultative roles, the critical importance of robust infrastructure and clear protocols, the necessity of framing AI within Saudi Arabia's family-centred care model, and the creative adaptive workarounds practitioners devised when encountering technical barriers. Chen et al. (36) (*n* = 12) contributed three further themes Potential of AI-Driven Nursing; Multi-Dimensional Response; and Obstacles to Intelligent Nursing highlighting both the aspirational promise of robotics and large-language–model support and the substantial challenges posed by funding constraints, systems immaturity, and the complexities of human–technology co-design.

Rony, Numan, Johra, et al. (54), (*n* = 37) advanced the conversation with a hermeneutic-phenomenological study that crystallized six ethical imperatives governance protocols, professional accountability, data privacy, algorithmic transparency, patient advocacy, and informed consent which participants aptly described as “protective walls” essential for sustaining patient trust and ensuring that AI augments rather than undermines the core values of nursing.

These qualitative insights resonate strongly with the quantitative findings: student fears of dehumanization mirror Lukić et al.'s (70) low practical-advantages subscale score (9.98/20 vs. neutral = 12), calls for robust governance echo widespread data-security concerns, and frontline accounts of leadership's catalytic role align with the 70% facilitator endorsement reported by Ramadan et al. (60).

### 6.5 Quality assessment

We conducted a comprehensive appraisal of methodological rigor across thirty-seven studies twenty-eight analytical cross-sectional surveys, one quasi-experimental trial, and eight qualitative inquiries using our predefined quality thresholds and checklists. Among the cross-sectional surveys, twenty-seven scored 7–8 on the JBI Analytical Cross-Sectional Checklist and were classified as high quality, while one scored 6 and was rated moderate quality (see Supplementary Appendix 3, Table S1). These high-quality studies uniformly demonstrated clear inclusion/exclusion criteria, thorough descriptions of settings and populations, use of validated or formally reliability-tested instruments, and appropriate statistical analyses (t-tests, ANOVA, chi-square, Pearson correlations). For example, Lukić et al. (70) reported a back-translated, Cronbach's α > 0.85 attitude scale and near-complete demographic characterization.

However, only twelve of the twenty-eight surveys implemented multivariable adjustment techniques to manage confounders—such as age, gender, academic year, and prior AI training—while the remaining fifteen relied solely on bivariate analyses. Studies such as El-Sayed et al. (64) and Al Omari et al. (50) which used linear regression or structural equation modeling, exemplify best practices in isolating predictor effects. One study (40) received a moderate quality rating precisely because of its lack of confounder control despite otherwise robust methods.

Turning to the eight qualitative inquiries, five scored ≥ 8 on the JBI Qualitative Checklist and were deemed high quality, while three scored 7 and were classified moderate quality (see Supplementary Appendix 3, Table S2). The high-quality studies exhibited exemplary alignment among research questions, philosophical stance, data collection, and analysis, as well as clear reflexivity and strong participant-voice representation. The two moderate-quality studies such as Summers et al. (65) underreported researcher positionality but nonetheless provided rich thematic depth.

The single quasi-experimental trial achieved a score of 8 out of 9 on the JBI Quasi-Experimental Studies Checklist, earning a high-quality classification (see Supplementary Appendix 3, Table S3). It demonstrated clear cause–effect linkage, comparable participant groups, and reliable outcome measurement, with only the absence of a randomized control arm limiting its design.

These results establish a solid foundation for synthesizing both thematic and quantitative findings, while indicating that confounder management remains the primary area for methodological improvement in future investigations of AI in nursing practice and education.

### 6.6 Risk of bias

We assessed internal validity across the same thirty-seven studies using six bias domains for quantitative work and five domains for qualitative work. “Overall Risk of Bias” reflects a synthesized judgment across those domains.

Across the twenty-eight cross-sectional surveys, selection bias was low in five studies (39, 64, 68, 69, 71) and moderate in the remaining twenty-three (see Supplementary Appendix 4, Table S1). Measurement consistency and detection bias were uniformly rated low risk across all surveys, given standardized administration protocols and validated instruments. Attrition bias was moderate in three studies (41, 59, 63) and low in the other twenty-five. Reporting bias was low across the board, as methods and results were transparently presented. Confounding bias was rated low in eight surveys and moderate in twenty. The overall risk of bias was low in two studies (64, 68) and moderate in the other twenty-six.

All eight qualitative inquiries were assessed against five JBI bias domains (see Supplementary Appendix 4, Table S2). Each exhibited moderate selection bias due to purposive sampling, with the remaining four domains rated low risk. Consequently, every qualitative study carried an overall moderate risk of bias, reflecting both the strengths and inherent limitations of thematic research in AI adoption among nursing populations. The quasi-experimental trial's risk-of-bias profile detailed in Supplementary Appendix 4, Table S3 showed low risk in intervention classification, participant adherence, outcome measurement, and reporting, but moderate risk in confounding and participant selection under ROBINS-I criteria, yielding an overall moderate risk of bias.

## 7 Discussion

This systematic review synthesizes evidence from diverse quantitative and qualitative studies to illuminate how nursing students and practicing nurses worldwide perceive and prepare for AI in healthcare. The findings reveal a cautiously optimistic outlook toward AI's potential benefits, tempered by persistent concerns about ethics, privacy, and the preservation of core nursing values. Attitudes towards AI in both groups are generally positive, with nursing students and clinical nurses recognizing AI's capacity to enhance efficiency and decision-making. However, these attitudes vary by demographics and experience: younger and more tech-savvy individuals tend to be more receptive, whereas some female and older nurses exhibit greater skepticism. AI literacy—defined as knowledge and technical competence regarding AI—is moderate on average but strongly linked with positive attitudes and willingness to adopt AI tools. Notably, individuals who have received AI training or exposure demonstrate significantly higher literacy and confidence. Behavioral readiness to integrate AI, reflected in intention-to-use, emerges from a complex interplay of cognitive (e.g., perceived usefulness), affective (trust and anxiety), and contextual (support and resources) factors, with theoretical models explaining variance in adoption intentions. Qualitative insights from nurses' and students lived experiences enrich these findings, underscoring fears that unbridled AI could erode critical thinking and compassionate care while emphasizing the enabling role of leadership, training, and ethical safeguards. This suggests that successful and sustainable AI integration in nursing hinges on boosting AI literacy and self-efficacy, fostering positive yet realistic attitudes, addressing valid ethical reservations, and strengthening organizational support structures. Our discussion will interpret these results in context, examine underlying determinants and barriers, evaluate the methodologies and frameworks employed, and propose an integrative model with actionable recommendations to guide education, practice, and policy for ethical and effective AI adoption in nursing.

## 8 Attitudes and perceptions: guarded optimism

These findings suggest that nursing stakeholders approach AI with a nuanced combination of cautious optimism and pragmatic concern. On one hand, consistently positive attitude scores whether around mid-60s on a 0–100 scale or above mid-3's on 5-point scales reflect a broad recognition of AI's capacity to streamline documentation, reduce medication errors, and bolster decision support (44, 55, 70). Such “guarded positivity” aligns with established technology-acceptance frameworks, in which perceived usefulness (performance expectancy) emerges as the primary driver of favourable attitudes. Yet, this optimism is held in check by tangible concerns: worries about diagnostic errors, data-privacy risks, infrastructure deficits, and insufficient training inhibit unqualified endorsement of AI (45, 53, 60).

In practice, this implies that demonstrations of AI's real-world efficacy must be accompanied by investments in robust technical systems, clear governance protocols, and user-centred design to convert initial enthusiasm into sustained engagement.

Importantly, demographic and experiential factors moderate these attitudes. Male and younger participants and those who have already engaged with AI through coursework or regular use tend to report higher literacy and more positive outlooks, suggesting that familiarity breeds confidence (63).

However, context matters: in some settings, such as the Alexandrian cohort studied by Hamad et al. (62), female students outpaced male peers once other variables were controlled, indicating that cultural and educational environments can invert expected gender patterns. Similarly, advanced-year students consistently display greater receptivity than juniors, underscoring the importance of longitudinal curricular integration. These nuances call for tailored strategies targeted workshops, mentorship by “AI champions”, and ongoing support to engage more skeptical subgroups (e.g., late-career nurses or those with limited prior exposure), ensuring equitable upskilling across the workforce.

Nursing professionals are broadly optimistic about AI's potential impact on care delivery, provided that robust validation, user-centred design, comprehensive training, and alignment with nursing's core values of empathy and patient advocacy are ensured. The absence of longitudinal designs, low response-rate reporting, and inconsistent demographic adjustments weaken our confidence in extrapolating these attitudes to diverse clinical contexts.

Beyond perceived usefulness at the bedside, clinicians' trust is shaped by system-level signals. Recent integrative evidence shows that fragmented regulation, uneven data governance, and interoperability issues can dampen frontline confidence even when perceived benefits are high (72). Clear accountability and transparency standards therefore operate as attitudinal enablers, not just compliance checkboxes (72).

## 9 AI literacy, digital self-efficacy, and readiness

Our findings suggest that nursing stakeholders possess a baseline familiarity with AI concepts yet lack the deep, consistent competence needed for confident clinical application. Moderate mean literacy scores alongside significant gains among students who have received dedicated AI coursework indicate that knowledge is acquirable but not uniformly distributed (63). The positive correlations between literacy and attitude underscore that improving factual understanding can foster more favorable dispositions; however, this relationship is neither automatic nor sufficient. In particular, the pronounced drop from immediate post-workshop proficiency (82.8%) to three-month retention (68.0%) in Egypt highlights the transience of one-off interventions and the need for longitudinal reinforcement (56).

Beyond technical know-how, readiness for AI integration depends critically on digital self-efficacy and emotional comfort. Path models show that positive attitudes and supportive environments drive intention to use AI, yet these attitudes themselves emerge from beliefs about performance expectancy, effort expectancy, and self-efficacy (68). Conversely, elevated AI anxiety as observed in neonatal nurses is a potent barrier, mediating the link between literacy and attitude (38). This dual cognitive–affective dynamic implies that educational strategies must pair robust, hands-on technical training with deliberate efforts to build confidence and allay fears. Simulations, mentorship programs, and iterative pilot projects offer promising avenues to normalize experimentation with AI tools, reinforce learning, and embed proficiency into routine practice.

Educational strategies must pair robust technical training with interventions to build digital self-efficacy and reduce anxiety through simulations, mentorship, and iterative pilot projects to achieve truly comprehensive readiness for AI integration in nursing. These findings reveal that while targeted education can yield rapid gains, sustained competency likely requires embedded, iterative training.

Readiness also hinges on whether organizations resource the full adoption lifecycle. Health-economic syntheses indicate many AI use-cases are cost-effective or cost-saving, but real-world value depends on accounting for training, integration, and maintenance costs that are often under-reported in primary studies (73). Programs that budget for these items alongside skills-building sustain literacy gains and actual use (73).

## 10 Determinants, barriers, and organisational facilitators

Our findings underscore that successful AI integration demands a multi-pronged strategy: cultivating individual digital fluency through scaffolded education, fortifying technical and governance infrastructures, and fostering an organizational ecosystem in which leadership actively champions AI as both a clinical asset and a professional development opportunity.

### 10.1 Individual-level determinants

Individual-level determinants of AI readiness in nursing extend beyond mere exposure to technology; they reflect deeper processes of professional identity formation and lifelong learning. Our synthesis suggests that as nursing students progress through their training, they not only accumulate technical knowledge but also develop the self-confidence and clinical judgment necessary to contextualize AI tools within patient care. This progressive attitudinal shift underscores the value of spiral curricula that revisit AI concepts at increasing levels of complexity pairing theoretical foundations in early years with applied, simulation-based experiences in later semesters. Embedding AI learning objectives into clinical practicums and capstone projects can transform passive familiarity into active competence, enabling nurses to critically evaluate algorithmic outputs rather than simply follow them.

### 10.2 Technical and governance barriers

Despite this potential, persistent technical and governance barriers threaten to undermine individual readiness by eroding trust and interrupting workflow. Nurses' reports of system downtime, slow interfaces, and siloed data reflect a disconnect between AI prototypes tested in controlled settings and the fragmented, high-stakes realities of clinical environments. Moreover, unresolved concerns about data privacy, algorithmic bias, and liability create psychological distance between practitioners and emerging technologies. Addressing these obstacles will require rigorous implementation science: multifaceted evaluations that link specific infrastructure investments such as interoperable platforms and real-time IT support to measurable improvements in safety, efficiency, and user satisfaction. Parallel efforts should engage nurses in the co-design of governance protocols, ensuring that policies around consent, accountability, and transparency resonate with frontline priorities.

### 10.3 Leadership and organisational enablers

Visible executive commitment characterised by dedicated funding, identification of “AI champions”, and clear communication of strategic priorities correlates with higher reported readiness among frontline nurses. In Saudi Arabia, 70% of respondents credited strong leadership support with fostering a culture open to experimentation (60). Nurse-led governance committees have been advocating establishing transparent ethical and legal protocols. Yet, no study to date has systematically examined whether tying AI competencies to performance metrics or incentive structures yields sustained improvements in adoption or clinical outcomes.

These insights call for a systemic, multilevel strategy: integrate AI education longitudinally to cultivate digital fluency; fortify technical and governance infrastructures through evidence-based implementation research; and sustain momentum with proactive, ethically grounded leadership. Only by aligning individual capabilities, organizational resources, and policy frameworks can we move from isolated pilot successes to pervasive, equitable integration of AI in nursing practice. Such a coordinated approach holds promise not only for optimizing patient outcomes but also for elevating nursing's role as a co-creator of the healthcare innovations of tomorrow.

Evidence published after the search date (June–August 2025).

Seven post-search studies extend our conclusions across distinct roles and settings without altering them materially (74–80). Nurse leaders and managers again showed higher perceived impact than preparedness with no correlation between the two constructs, and lower “impact” among BSN vs. MSN and in medical departments underscoring that impact and preparedness have partly distinct drivers (80). Turkish nurse managers framed AI as a strategic tool for safety, quality, documentation, medication management, and workforce planning, but insisted on governance-first, pilot-driven deployment, funded infrastructure, and continuous training (77). Among U.S. nursing faculty, attitudes were neutral-to-positive despite minimal–moderate self-rated knowledge/skills; doctoral preparation and the presence of AI policy/syllabi language aligned with higher knowledge suggesting that faculty development and clear policy signals are readiness multipliers (78).

In education, Palestinian BSN students reported high AI anxiety coexisting with positive attitudes; anxiety was higher among female students, non-users, and younger learners, marking priority subgroups for reassurance and scaffolded exposure (76). In Egypt, more positive AI attitudes tracked strongly with creative self-efficacy and clinical reasoning, and training/qualifications associated with stronger competencies linking pro-AI mindsets to core clinical capabilities (74). In Iranian hospitals, knowledge, attitude, acceptance, and application were tightly inter-correlated; application was jointly predicted by all three, while head-nurse role predicted lower application, pointing to role-specific workflow/liability barriers at the leadership interface (75). Finally, Bangladeshi nurse-educators emphasized benefits for engagement and personalization but highlighted training deficits, infrastructure/connectivity gaps, and ethical governance as conditions for success (79).

Overall, these studies strengthen our main pattern perceived benefits outpacing readiness, with role- and policy-linked gradients and sharpen implications: invest in governance and infrastructure, faculty development and syllabi policy, targeted anxiety-reducing skills-building for vulnerable subgroups, and role-tailored enablement for nurse leaders/managers. Because they were identified after our prespecified cutoff, we synthesize them narratively here and do not include them in counts or pooled analyses.

### 10.4 Methodological and theoretical perspectives

Our review reveals a predominant reliance on cross-sectional, single-timepoint designs, with sample sizes ranging from approximately 107 to 1 713 participants and heavy dependence on convenience sampling. This design choice, while expedient, constrains internal validity, prevents causal inference, and obscures temporal dynamics notably, how nursing students' and practicing nurses' attitudes, literacy and anxiety toward AI evolve as integration deepens in curricula and clinical practice. The solitary quasi-experimental investigation hints at the promise of targeted educational interventions (56) but the absence of longitudinal follow-ups represents a pivotal gap in our understanding of sustained attitude change and skill acquisition over time.

Measurement practices likewise exhibit marked variability. Although flagship instruments such as the Medical AI Readiness Scale (α = 0.934), Artificial Intelligence Anxiety Scale (α = 0.942) and the Scale for the Assessment of Nonexperts' AI Literacy (α = 0.93) demonstrate robust internal consistency, a substantial subset falls below psychometric benchmarks (AILS α = 0.766; GAAIS α = 0.792) or lacks any reported validity or reliability data, even after adoption (36, 37, 41, 48, 57). Moreover, half of the 28 surveys reutilized existing tools without full re-validation in local nursing contexts, raising concerns about construct drift and response bias. Without rigorous psychometric re-evaluation, comparisons across studies and any subsequent meta-analytic synthesis remain precarious (37, 39, 42–44, 48, 49, 57, 59, 64, 66, 67, 69, 71).

Theoretical anchoring across quantitative work is highly fragmented, with only a minority of studies grounding their models in established frameworks. Seven surveys explicitly drew on TAM, UTAUT or UTAUT2 (35, 43, 49, 62, 67–69). Two studies explicitly referenced the Theory of Reasoned Action (TRA) and the Theory of Planned Behavior (TPB) (69, 70). Only one incorporated a dual lens of social cognitive and self-determination theories (64). The sporadic integration of Diffusion of Innovations alongside TAM (62) and the near silence of qualitative ethical and normalization insights (60, 65) underscore a broader conceptual disunity. This fragmentation complicates efforts to identify coherent determinants of AI adoption and impedes accumulation of cumulative knowledge.

Analytic sophistication remains the exception rather than the rule. Nine surveys progressed beyond basic descriptive statistics employing path analyses in AMOS (66, 68), mediation and moderation testing via Hayes' PROCESS macro (49, 64, 67, 71), two performed factor analyses; Kotp et al. (47) conducted both exploratory and confirmatory factor analyses; Tsiara et al. (42) conducted confirmatory factor analysis, and one full structural equation model with fit indices (43). These advanced methodologies have begun to illuminate latent relationships among constructs, yet their limited deployment restricts our capacity to delineate causal pathways and to test complex, theory-driven hypotheses.

### 10.5 Conceptual framework for AI adoption in nursing

Drawing on our thematic synthesis, we propose a multi-layered conceptual framework that captures the complex interplay of individual, technological, organizational, and policy factors shaping AI adoption in nursing.

This multi-layered framework is hypothesis-generating: it is derived from synthesis rather than tested empirically. Prospective validation via longitudinal or experimental designs linking literacy, attitudes, anxiety, and facilitating conditions to observed uptake and safety outcomes is needed.

At its core lies knowledge–Attitude–Intention trajectory, whereby foundational AI literacy and digital self-efficacy engender positive attitudes, which in turn form the cognitive substrate for adoption intentions. These individual-level processes are modulated by affective constructs namely anxiety and trust underscoring that cognitive competence alone is insufficient without addressing emotional readiness. Surrounding this core, we delineate two concentric contextual strata: (1) Facilitating Conditions, encompassing infrastructure quality, leadership advocacy, resource allocation, and peer support; and (2) Ethical & Cultural Governance, covering data privacy protocols, liability frameworks, bias-mitigation strategies, and alignment with professional nursing values. Each layer exerts both direct and indirect influences, such that deficiencies in technological usability or poor change-management practices can attenuate even the most robust individual readiness, while clear ethical guidelines and co-designed interfaces can not only bolster trust but also translate intention into sustained practice. Importantly, our model refrains from presuming causal direction in existing cross-sectional associations, highlighting the urgent need for empirical validation through longitudinal and experimental studies to confirm the directional pathways and magnitudes of these relationships.

### 10.6 Actionable recommendations and future research directions

Building on this framework, we propose targeted strategies to guide curriculum design, faculty development, organizational policy, and research priorities. First, nursing curricula must integrate AI competencies; technical concepts, ethical reasoning, and hands-on simulations progressively across undergraduate and graduate programs to reinforce knowledge, self-efficacy, and reflective practice. Faculty development initiatives should leverage interdisciplinary workshops and mentorship by AI experts so educators can model responsible technology use. In clinical settings, institutions should appoint nurse AI champions, ensure seamless EHR integration, and adopt inclusive change-management processes that solicit frontline feedback. Concurrently, professional associations and ethics committees must co-develop clear guidelines on informed consent, accountability, data governance, and bias mitigation, thereby creating a normative environment for ethically sanctioned AI use. Equity-focused policies such as targeted funding and infrastructure grants for under-resourced or rural settings are essential to prevent AI integration from widening existing disparities in nursing education and patient care. In parallel, governance and standards must be treated as core readiness enablers: co-design with nurses, adopt shared data/metadata standards, and mandate real-world performance monitoring with unambiguous accountability pathways. Institutions should also plan explicitly for total cost of ownership by budgeting for training, integration, updates, and IT support, and by reporting both cost-effectiveness and budget impact including subgroup and equity analyses to avoid unintended disparities.

To strengthen the evidence base and refine this conceptual model, future research should employ longitudinal or experimental designs that capture trajectories of AI adoption and disentangle causal pathways; conduct cross-cultural validation and psychometric standardization of core AI literacy, attitude, and readiness instruments; implement mixed-method trials linking self-reported measures with objective behavioral outcomes such as usage logs and patient-safety indicators to assess real-world impact; and engage deeply with ethical, organizational, and socio-cultural theories (e.g., CFIR) to guide sustainable integration. By prioritizing methodological rigor and theoretical integration, the nursing community can validate the proposed framework and proactively shape the profession's digital evolution, ensuring that AI augments compassionate care rather than supplants it.

It is important to note that our proposed multi-layered conceptual framework and corresponding recommendations have not been directly tested in any primary study included in this review. They are intended as an exploratory synthesis of cross-sectional associations and qualitative insights rather than empirically validated interventions. Future research should empirically evaluate each element of this framework through longitudinal, experimental, or mixed-methods studies before adoption in policy or curricula.

### 10.7 Strengths & limitations

This systematic review's major strengths lie in its rigorous, transparent methodology following PRISMA 2020, dual independent screening (κ = 0.78 and κ = 0.74), and use of the SPIDER framework and its comprehensive scope, which combines 28 analytical surveys, one quasi-experimental trial, and eight qualitative studies. Grounding analyses in established models (TAM, UTAUT/UTAUT2, TRA/TPB) and employing thematic synthesis, standardized quality appraisals (JBI checklists, ROBINS-I), and subgroup comparisons of psychometric tools (e.g., GAAIS, AILS, MAIRS-MS) enhances both validity and interpretability. Actionable recommendations and a proposed conceptual framework further translate findings into practice and policy.

By summing JBI scores into a single rating, domain-specific nuances may be obscured. Limiting inclusion to English-language, peer-reviewed articles and excluding gray literature introduces potential language and publication biases. The narrative synthesis of heterogeneous designs and measures precluded statistical heterogeneity testing (e.g., *I*^2^), while the geographic concentration in Turkey, Egypt, and Saudi Arabia limits global generalizability. Only 43% of cross-sectional surveys used multivariable adjustment (with confounding bias rated low in 8 studies and moderate in 20, and overall risk of bias low in 2 and moderate in 26), leaving residual confounding that tempers our overall quality judgment to “moderate” rather than uniformly “high”.

A principal limitation is uneven global coverage: studies cluster in a narrow set of national contexts and delivery settings, constraining external validity. We therefore bound inference to settings with similar digital readiness, governance maturity, and educational capacity, and we explicitly avoid claims of universal generalisability.

## 11 Conclusion

This systematic review demonstrates that nursing students and nurses possess optimistic attitudes toward AI, underpinned by moderate literacy and readiness yet tempered by infrastructure, ethical, liability concerns. We identified perceived usefulness, self-efficacy, and facilitating conditions as robust predictors of both attitudes and behavioral intentions, while AI-related anxiety and demographic moderators such as gender, seniority, and prior exposure influence these relationships. Critically, organizational support from visible leadership commitment to modernized IT infrastructure and comprehensive ethical and governance frameworks emerged as facilitators; conversely, outdated systems, data-privacy fears, and inconsistent training persist as barriers. To translate optimism into integration, stakeholders should embed AI competencies in curricula, augment simulations with workshops, and appoint nurse AI champions to co-design tools. Policymakers and bodies should codify ethical guidelines, ensure equitable resource allocation, and incentivize pilot programs. By aligning individual, technological, organizational, and policy strategies, nursing can harness AI to enhance patient safety, workflow efficiency, and excellence.

## Data Availability

The original contributions presented in the study are included in the article/Supplementary Material, further inquiries can be directed to the corresponding author.
