# Supplementary material for: Artificial intelligence in nursing: a systematic review of attitudes, literacy, readiness, and adoption intentions among nursing students and practicing nurses
Source: Front Digit Health. 2025 Sep 25;7:1666005. doi: 10.3389/fdgth.2025.1666005 (PMC12507812; doi:10.3389/fdgth.2025.1666005)
Supplement: Supplementary file 1 [file Datasheet1.pdf]

Supplementary Material 1. Database Search Strategy

Database	Search Strategy	Filters
**PubMed/MEDLINE**	**Concepts:** “Artificial Intelligence” OR “Machine Learning” OR “Deep Learning” OR “Neural Network*” OR “Natural Language Processing” OR ChatGPT OR “large language model*”<br>AND **Nursing terms:** nurs* OR “nursing student*” OR “registered nurse*” OR “nurse leader*” OR “nurse educator*”<br>AND **Acceptance terms:** attitude* OR perception* OR literacy OR readiness OR “behavioral intention” OR acceptance OR UTAUT OR UTAUT2 OR TAM	English; Humans
**Embase**	**Concepts:** ‘artificial intelligence’/exp OR ‘machine learning’/exp OR ‘deep learning’/exp OR ‘neural network’/exp OR ‘natural language processing’/exp OR title/abstract AI terms<br>AND **Nursing terms:** 'nurse'/exp OR 'nursing student'/exp OR title/abstract nurse terms<br>AND **Acceptance terms:** 'attitude'/exp OR 'perception'/exp OR title/abstract acceptance terms	English; Humans
**CINAHL**	**Concepts:** MH “Artificial Intelligence+” OR TI/AB AI terms<br>AND **Nursing terms:** MH “Nurses+” OR MH “Nursing Students+” OR TI/AB nurse terms<br>AND **Acceptance terms:** MH “Attitude” OR TI/AB acceptance terms	English; Academic journals
**Scopus**	TITLE-ABS-KEY(AI terms)<br>AND TITLE-ABS-KEY(nurse terms)<br>AND TITLE-ABS-KEY(acceptance terms)	English
**Web of Science**	TS=(AI terms)<br>AND TS=(nurse terms)<br>AND TS=(acceptance terms)	English; Articles
**IEEE Xplore**	Full-text search: AI terms AND nurse terms AND acceptance terms	Journals; English

Key term groups

- **AI terms:** "artificial intelligence," "machine learning," "deep learning," "neural network*," "natural language processing," ChatGPT, "large language model*"
- **Nurse terms:** nurs*, "nursing student*," "registered nurse*," "nurse leader*," "nurse educator*"
- **Acceptance terms:** attitude*, perception*, literacy, readiness, "behavioral intention," acceptance, UTAUT, UTAUT2, TAM, "Technology Acceptance Model"
