# Supplementary material for: Artificial intelligence in nursing: a systematic review of attitudes, literacy, readiness, and adoption intentions among nursing students and practicing nurses
Source: Front Digit Health. 2025 Sep 25;7:1666005. doi: 10.3389/fdgth.2025.1666005 (PMC12507812; doi:10.3389/fdgth.2025.1666005)
Supplement: Supplementary file 2 [file Datasheet2.pdf]

Supplementary Material 2. Characteristics of Included Studies

Study (Author, Year, Country)	Design & Setting	Population (N)	Measures / Instruments	Analysis & Main Findings
Lukić et al., 2023; Croatia<br><br>(Lukić et al., 2023)	Cross-sectional, multicenter study at four Croatian undergraduate nursing programmes	336 first-year nursing students)	Modified 20-item General Attitudes towards Artificial Intelligence Scale (GAAIS), five-point Likert, contextualized for nursing	Exploratory factor analysis (four factors)<br>Descriptive statistics, one-sample t-tests vs neutral, one-way ANOVA, Pearson correlation.<br>Found slightly positive overall attitude (64.5 ± 11.7 vs neutral 60, $p<0.001$), favourable "benefits", "willingness" and "dangers" subscales, but unfavourable "practical advantages" subscale.
Yalcinkaya T et al., 2024; Turkey<br><br>(Yalcinkaya et al., 2024)	Descriptive cross-sectional study at the Faculty of Nursing, state university, west Turkey	291 undergraduate nursing students (48 1st-year; 89 2nd-year; 83 3rd-year; 71 4th-year)	Individual Information Form:<br>General Attitudes towards AI Scale (GAAIS, Turkish version; 20 items, 2 subscales)<br>Medical AI Readiness Scale for Medical Students (MAIRS-MS; 22 items, 4 subscales)	Descriptive statistics and reliability (Cronbach's $\alpha > 0.90$); normality via skewness/kurtosis; inferential: one-sample t-tests against neutral, independent t-tests, one-way ANOVA with Bonferroni post-hoc, Pearson/Spearman correlations.<br>Found moderately positive attitudes and readiness; 4th-year students had significantly higher readiness than 2nd-years ($F = 3.75$; $p = 0.011$); positive attitude–readiness correlation ($r = 0.330$; $p < 0.01$).
Kwak, Seo, et al., 2022; Republic of Korea<br><br>(Kwak, Seo, et al., 2022)	Cross-sectional survey with path analysis at two nursing schools in Gyeonggi and Jeonnam Provinces, Republic of Korea	210 nursing students 183 (87.1 %) were female and 27 (12.9 %) were male	Four-item scales for performance expectancy, effort expectancy, social influence, facilitating conditions (UTAUT)<br>Self-efficacy (4 items) and anxiety (4 items; Social Cognitive Theory)<br>Behavioral intention (3 items; UTAUT)<br>General Attitudes towards Artificial Intelligence Scale (20 items: 12 positives, 8 negative)	Descriptive statistics; tests of normality (skewness/kurtosis) and multicollinearity (VIF, tolerance); Pearson's correlation<br>Path analysis via SPSS 26.0 and AMOS 26.0; model fit (GFI=0.956, SRMR=0.057, CFI=0.918, PNFI=0.152, ECVI=0.609)<br>Positive attitude ($\beta=0.485$, $p=0.009$) and facilitating conditions ($\beta=0.117$, $p=0.045$) directly predicted intent to use; performance expectancy, self-efficacy, and effort expectancy predicted positive attitude; performance expectancy and self-efficacy negatively predicted negative attitude while anxiety positively predicted it; social influence non-significant
Labrague LJ et al., 2023; Philippines (Labrague et al., 2023)	Cross-sectional study at two government-owned nursing schools in Eastern Visayas, Philippines	200 undergraduate student nurses (157 female [78.5%], 43 males [21.5%])	Attitudes towards AI Technology Scale (12 items; 5-point Likert)<br>AI Utilization in Nursing Practice Scale (modified; 5-point Likert)<br>Intentions to Use AI Technology Scale (3 items; 5-point Likert)	Descriptive statistics; Pearson's correlation; independent t-tests; one-way ANOVA<br>Mediation analysis via Hayes' PROCESS macro (Model 4)<br>Perceived AI utilization directly predicted attitudes ($\beta = 0.450$, $p < 0.001$) and intention to adopt AI ($\beta = 0.458$, $p < 0.001$); attitudes partially mediated this relationship (indirect $\beta = 0.255$; 95% CI 0.173–0.343)
Demir-Kaymak et al., 2024; Turkey	Relational survey at midwifery and nursing	480 students (240 nursing, 240	Personal information form:<br>Medical Artificial Intelligence	Descriptive statistics; inferential (independent t-tests, one-way ANOVA, Pearson correlations); path analysis via SPSS

(Demir-Kaymak et al., 2024)	departments, Sakarya University, Sakarya, Türkiye	midwifery; 25 male [5.2 %], 455 female [94.8 %]; aged 18–42)	Readiness Scale (22 items, four factors; Cronbach's α = 0.934)<br>• Artificial Intelligence Anxiety Scale (21 items, four factors; Cronbach's α = 0.93)	26.0 and AMOS 26.0. Model 1 showed readiness did not significantly predict anxiety (β=0.093, p=0.080). Model 2 (χ^2/df=2.994; CFI=0.947; RMSEA=0.065) found that AI knowledge (β=–0.163, p<0.001) and daily AI use (β=–0.117, p=0.015) predicted readiness; AI trust (β=0.161, p=0.002), perceived occupational threat (β=–0.183, p<0.001), and daily AI use (β=0.135, p=0.009) predicted AI anxiety .
Summers et al., 2024; Australia<br><br>(Summers et al., 2024)	Qualitative descriptive study with semi-structured interviews at the School of Health, Discipline of Nursing, University of the Sunshine Coast, Australia	13 nursing students (7 female [53.8%], 6 males [46.2%]; 10 domestic, 3 international; 5 first-year, 8 second-year)	Semi-structured interviews, digitally recorded and verbatim transcribed	Thematic analysis using Braun & Clarke's six-phase process; independent coding and team consensus. Six themes emerged, revealing more unfavourable than favourable views of generative AI, recognition of benefits in non-patient-facing tasks, concerns over patient-interaction and academic integrity, generational attitude differences, and recommendations for ethical integration and further research
El-Sayed et al., 2025; Egypt<br><br>(El-Sayed et al., 2025)	Descriptive, cross-sectional study with moderation analysis at the College of Nursing, Alexandria University, Egypt	596 undergraduate nursing students (after removal of 4 extreme responses)<br>39.4 % were male (n ≈ 235) and 60.6 % were female (n ≈ 361)	• Innovative Thinking Competencies Scale (17 items; observing, questioning, idea networking, experimenting; α = 0.90)<br>• Scale for the Assessment of Nonexperts' AI Literacy (31 items; technical understanding, critical appraisal, practical application; α = 0.93)<br>• Career and Talent Development Self-Efficacy Scale (18 items; talent development, work habits & values, career exploration; α = 0.92)	Descriptive statistics showed moderate scores on all scales. Pearson correlations indicated CTSE was positively associated with both innovation mindset (r = 0.546, p < 0.01) and AI literacy (r = 0.568, p < 0.01). Moderated linear regression (PROCESS macro v4.2) revealed significant positive effects of innovation mindset (B = 0.154, p = 0.003) and AI literacy (B = 0.221, p < 0.001) on CTSE, with a significant interaction (B = 0.074, p = 0.004), indicating that higher AI literacy strengthens the innovation mindset–CTSE relationship.
Akca Sumengen et al., 2025; Turkey<br><br>(Akca Sumengen et al., 2025)	Descriptive, correlational, cross-sectional online survey at the Faculty of Nursing, Yeditepe University, Istanbul	205 undergraduate nursing students (180 female [87.8 %], 25 males [12.2 %])	Participant Introduction Form<br>Artificial Intelligence Literacy Scale (AILS; 12 items, four sub-dimensions: awareness, usage, evaluation, ethics; 7-point Likert)<br>General Attitudes Towards Artificial Intelligence Scale (GAAIS; 20 items, positive and negative subscales; 5-point Likert)	Descriptive statistics for sociodemographic, scale scores; Kolmogorov–Smirnov confirmed normality<br>Student's t-tests and one-way ANOVA (with Bonferroni post-hoc) to compare groups<br>Pearson correlations between AILS and GAAIS scores<br>Findings: male students and those with prior AI education or regular AI use had significantly higher AILS and positive GAAIS scores (p<0.001); students perceiving higher income had greater AI literacy (p<0.05); third- and fourth-year students scored higher on AILS usage and attitudes (p<0.05);

				positive correlations between literacy and positive attitudes (r=0.318–0.519, p<0.001) and negative correlations with negative attitudes (r=–0.138 to –0.215, p<0.05)
Sarman & Tuncay, 2025; Turkey<br><br>(Sarman & Tuncay, 2025)	Cross-sectional descriptive study at a single university's Faculty of Health Sciences in eastern Turkey; in-person survey conducted in common areas (library, cafeteria) outside classrooms	314 undergraduate nursing students (210 female [66.9%], 104 males [33.1%])	Socio-demographic form<br>Artificial Intelligence Literacy Scale (AILS; 12 items, 4 dimensions; Cronbach's α=0.766)<br>General Attitudes Towards Artificial Intelligence Scale (GAAIS; 20 items; α=0.792)<br>Artificial Intelligence Anxiety Scale (AIAS; 16 items; α=0.942)	Descriptive statistics; normality tests (skewness/kurtosis); inferential: t-tests, ANOVA, Mann-Whitney U, Kruskal-Wallis H, Pearson/Spearman correlations; mediation analysis via Hayes' PROCESS macro in R<br>88.5% reported knowing AI; 63.7% aware of ChatGPT; 73.6% used AI<br>AI literacy correlated positively with attitudes (r=0.433, p<0.01) and with anxiety (r=0.322, p<0.01)<br>Mediation analysis showed AI anxiety partially mediated the effect of AI literacy on general attitudes (indirect effect's 95% CI excluded zero)
Hamad et al., 2025; Egypt<br><br>(Hamad et al., 2025)	Descriptive cross-sectional survey at the Faculty of Nursing, Alexandria University, Egypt	600 undergraduate nursing students included (after attrition), 419 (69.8 %) were female and 181 (30.2 %) were male	General Attitudes Towards Artificial Intelligence Scale (20 items; positive/negative subscales)<br>Technology Acceptance Model questionnaire (12 items; perceived usefulness & ease of use)<br>Demographic and AI-experience form	Descriptive statistics; independent t-tests; one-way ANOVA; Pearson correlations (GA–PU r = 0.423, p < 0.001);<br>Linear regression identifying predictors of GA (female B = 2.950, p < 0.001; prior AI training B = 3.012, p < 0.001; "friends" as info source B = –1.979, p = 0.005; academic level B = 0.602, p = 0.024) and of PU (academic level B = 0.528, p < 0.001; AI training B = 1.630, p < 0.001; female B = 1.191, p < 0.001; "internet" as info source B = –1.265, p = 0.021)
Rony, Kayesh, et al.,2024; Bangladesh<br><br>(Rony, Kayesh, et al., 2024)	Descriptive qualitative study at three tertiary-level private hospitals in Dhaka city, Bangladesh	23 nursing professionals (purposively sampled senior staff nurses, charge nurses, nurse managers, lecturers; 10 domestic, 3 international)	Semi-structured interviews (audio-recorded, verbatim transcribed)	Thematic analysis (Braun & Clarke's six-phase process) with line-by-line coding, constant comparative method, independent coding and team consensus; identified ten key themes showing a balance of optimism and caution, highlighting needs for training, ethical safeguards, and a symbiotic AI–nurse partnership to enhance patient-centred care
Ramadan et al., 2024; Saudi Arabia<br><br>(Ramadan et al., 2024)	Descriptive qualitative study using focus groups at four major healthcare facilities in Al-Kharj (general, defence, maternal–	48 registered nurses (37 female [77.1 %], 11 males [22.9 %]; mean age 36.7 ± 8.4 y,	Semi-structured focus-group interview guide (domains: AI understanding; facilitators; barriers; attitudes)<br>Demographic questionnaire<br>Field-notes template	Thematic analysis (Braun & Clarke six-phase): identified 4 themes and 12 subthemes (understanding, facilitators, barriers, attitudes). Participants held mainly positive yet cautious views of AI, emphasising the need for leadership commitment, user-centred design, robust training, ethical safeguards, and infrastructure upgrades. Proposed an

	child, and university hospitals)	mean experience 11.3 ± 7.2 y)		extended TAM-AIN framework incorporating ethical alignment, organisational readiness, professional identity preservation, and technical capacity .
Alruwaili et al., 2024; Saudi Arabia<br><br>(M. M. Alruwaili et al., 2024)	Descriptive cross-sectional design at three governmental hospitals (King Abdulaziz, Prince Mut'eb, Domat Al-Jandal) in Jouf region, northern Saudi Arabia	220 registered nurses	Demographic questionnaire<br>Nurses' AI knowledge questionnaire (7 items, yes/no; Swed et al.)<br>General Attitudes Towards AI Scale (GAAIS; 20 items; 5-point Likert; Schepman & Rodway)	Descriptive statistics (frequencies, percentages; means ± SD); χ^2 tests for associations:<br>– Moderate awareness (58.2–70.9%) and moderate attitudes (item means 2.87–3.66)<br>– Female nurses exhibited more negative attitudes (χ^2=4.67, p=0.03)<br>– Younger nurses more positive (χ^2=9.31, p=0.02)<br><br>– No significant effects of marital status or years' experience
Almagharbeh et al., 2025; Jordan<br><br>(Almagharbeh et al., 2025)	Exploratory descriptive qualitative study using semi-structured interviews (n=25) and three focus-group discussions (7–8 participants each) with registered nurses in public and private healthcare facilities across Amman, Jordan	25 nurses (individual interviews) and 3 focus groups of 7–8 nurses each (total FG participants 21–24)	Semi-structured interview guide<br>Focus-group discussion guide	Thematic analysis via Braun & Clarke's six-phase method using NVivo 14; independent coding with team consensus. Three overarching themes emerged: (1) AI as an enabler of efficiency; (2) ethical and practical challenges; (3) need for structured training and education to support safe, effective AI integration
Abou Hashish & Alnajjar, 2024; Saudi Arabia (Abou Hashish & Alnajjar, 2024)	Descriptive correlational, cross-sectional survey at College of Nursing-Jeddah, King Saud bin Abdulaziz University for Health Sciences, Jeddah (female-only)	266 third- and fourth-year undergraduate nursing students (all female	Structured six-section questionnaire:<br>Personal information form<br>Knowledge of Digital Transformation (19 items)<br>Attitudes toward Digital Transformation (11 items)<br>Digital Skills (6 items)<br>Digital Health Literacy (8 items)<br>Attitudes toward AI (5 items)<br>All on 5-point Likert scales	Descriptive statistics; Student's t-test and one-way ANOVA for academic-level comparisons; Pearson correlation:<br>– High digital transformation knowledge, skills, and DHL; moderate positive ATD and AAI<br>– Knowledge significantly correlated with ATD, DS, DHL, AAI ($p < 0.001$)<br>– 4th-year students scored higher than 3rd-years on knowledge, DS, and DHL ($p < 0.05$) but not on ATD or AAI<br>– Key facilitators: reliable internet, tool availability, public awareness; barriers: user knowledge gaps, resource constraints
Hussein Mohamed et al., 2023; Egypt	Quasi-experimental pre–post intervention study in medical and surgical departments at Mansoura University Hospitals	203 staff nurses (180 female [88.7 %], 23 male [11.3 %])	Self-administered AI Knowledge Questionnaire (12 domains; true/false; scored 0–2 per item)<br>General Attitudes towards AI Scale (24 items; 5-point Likert;	Descriptive statistics; chi-square for knowledge items; one-way ANOVA for attitude items<br>Knowledge "satisfactory" rose from 16.3 % pre-intervention to 82.8 % immediate post (all $\chi^2 > 125$, $p < 0.001$) and declined to 68.0 % at 3-month follow-up<br>Positive attitude increased from 26.1 % pre to 81.8 % post (F

(Hussein Mohamed et al., 2023)			adapted from Schepman & Rodway)	= 65.2, p < 0.001) and fell to 63.5 % at follow-up<br>Educational program had highly significant positive effects on both knowledge and attitudes across all domains.
Ahmed et al., 2024; Egypt<br><br>(Ahmed et al., 2024)	Exploratory research design at Minia University Hospital, a pediatric–obstetric university hospital, and a kidney disease & urology hospita	455 staff nurses in 254 (55.8 %) were female and 201 (44.2 %) were male	Perception toward AI questionnaire (adapted from Abdullah & Fakieh 2020): 4 dimensions—perception (4 items), benefits (5 items), implementation challenges (5 items), perceived barriers (7 items); 5-point Likert<br>General Attitudes toward AI questionnaire (adapted from Schepman & Rodway 2020): 20 items; 5-point Likert	Descriptive statistics; independent t-tests and one-way ANOVA to compare groups; Spearman correlation between perception and attitude. Found moderately positive perception and attitude toward AI; highest perception for "AI reduces medical errors" (4.04 ± 0.96); no significant differences by hospital, age, gender, or residence, while education level and <15 years' experience showed higher scores (P = 0.018); strong positive correlation between perception and attitude (r = 0.715, P < 0.001)
Rony, Numan, Johra, et al., 2024; Bangladesh<br><br>(Rony, Numan, Johra, et al., 2024)	Descriptive, phenomenological qualitative study using in-depth interviews at five tertiary-level health care institutions in Dhaka, Bangladesh	37 nurse practitioners	Semi-structured interview guide; face-to-face or video-conference interviews (50–60 min), audio-recorded and verbatim-transcribed	Thematic analysis via Braun & Clarke's six-phase process, with member checking and peer debriefing; uncovered a balanced mix of optimism and caution, highlighting AI's potential to enhance patient care, the imperative for ethical safeguards, structured training, and collaborative frameworks for responsible integration
Salama et al., 2025; Palestine<br><br>(Salama et al., 2025)	Cross-sectional survey at eight private and governmental universities in the West Bank, Palestine, using an online self-administered questionnaire via Google Forms	304 nursing students (215 female [70.7 %], 89 male [29.3 %])	Multi-section KAP questionnaire:<br>Demographics<br>AI knowledge (definitions, machine learning, applications)<br>Attitudes toward AI and ChatGPT (benefits, concerns, integration)<br>Practices of AI and ChatGPT use	Descriptive statistics (frequencies, percentages, mean ± SD), chi-square tests, independent t-tests and one-way ANOVA (p<0.05); revealed high AI awareness (84.5 %) but limited formal education (41.8 %), strong support for curricular inclusion (79 %) yet concerns about errors (62.2 %), moderate AI use (58.6 %) and low ChatGPT adoption (33.9 %), with significant differences by gender, age and academic year
Kahraman et al., 2025; Türkiye<br><br>(Kahraman et al., 2025)	Descriptive cross-sectional online survey of perioperative nurses across surgical units in Türkiye, using snowball sampling via mobile, email, and social media	505 perioperative nurses (399 female [79.0 %], 106 male [21.0 %]	• Nurse Information Form (demographics; AI training, awareness, use; perceived workload impact; IT proficiency)<br>• AI Literacy Scale (12 items; four subdimensions—Awareness, Use, Evaluation, Ethics; 7-point Likert; Cronbach's α = 0.821)	Descriptive statistics (means, SDs, frequencies); normality via skewness/kurtosis<br>Inferential: independent t-tests, dependent t-tests, one-way ANOVA, Pearson correlations (p<0.05)<br>Male nurses scored significantly higher on Awareness, Use, Evaluation, and total literacy (p<0.05)<br>Nurses who had heard of or used AI, who believed AI reduces workload, and with higher IT proficiency had higher literacy (p<0.05)

				Age inversely correlated with literacy (r=–0.179 to –0.061; p<0.05)
Rony, Numan, Akter, et al., 2024 Bangladesh<br><br>(Rony, Numan, Akter, et al., 2024)	Hermeneutic phenomenology qualitative study at four tertiary hospitals in Dhaka; purposive sampling of registered nurses with ≥ master's degree and semi-structured interviews	20 registered nurses (12 female [60 %], 8 male [40 %]; age 28–52 y, mean 37.2 y)	Semi-structured interview guide (validated via pilot and expert review); 50–60 min audio-recorded, verbatim-transcribed individual interviews in Bangla	Thematic analysis using Braun & Clarke's six-phase process within a hermeneutic phenomenological framework, with member checking and an audit trail; revealed nurses' emphasis on robust ethical safeguards, data privacy, human-centred AI adoption, and the need for structured training and organizational support
Al Omari et al., 2024; Bahrain, Egypt, Iraq, Jordan, Kuwait, Lebanon, Oman, Palestine, Saudi Arabia, UAE<br>(Al Omari et al., 2024)	Descriptive cross-sectional online survey via convenience sampling across 10 Arab countries	1 713 nursing students (mean age 22.45 ± 3.56 y; 1 183 female [69.1 %], 530 male [30.9 %])	Demographic form<br>Intention to use AI scale (3 items, 5-point Likert; α = 0.873)<br>Knowledge of AI scale (8 items, 3-point Likert; α = 0.811)<br>Perception of AI scale (10 items, 5-point Likert; α = 0.885)<br>Attitude towards AI scale (3 items, 5-point Likert; α = 0.890)	Descriptive statistics; bivariate (t-tests, ANOVA); Pearson correlations; multiple linear regression (final model F = 126.283, p < 0.001, R^2 = 0.342): perception (β = 0.295), attitude (β = 0.211), knowledge (β = 0.061), age (β = 0.074), AI understanding (β = 0.092), self-rated tech-savviness (β = 0.108), clinical performance (β = 0.060) all independently predicted intention to use AI
Cho & Seo, 2024; South Korea<br><br>(Cho & Seo, 2024)	Descriptive, cross-sectional study at two nursing schools in Gyeonggi Province and Gwangju Metropolitan City	180 nursing students, 26 (14.4 %) were male and 154 (85.6 %) were female .	Shinners Artificial Intelligence Perception tool (10 items; preparedness & professional impact subscales; 5-point Likert)<br>Anxiety about AI scale (4 items; 5-point Likert; adapted from TAM)<br>Acceptance attitude toward AI scale (4 items; 5-point Likert; TAM)<br>Intention to use AI scale (3 items; 5-point Likert; TAM)	Descriptive statistics: means, SDs; independent t-tests, one-way ANOVA; Pearson correlations<br>Mediation analysis via PROCESS Macro model 6 (10 000 bootstrap samples): perception negatively predicted anxiety (b = –0.420, p < 0.001) and positively predicted acceptance attitude (b = 0.541, p < 0.001); acceptance attitude (b = 0.246, p < 0.001) and perception (b = 0.280, p = 0.002) directly predicted intention to use AI; total indirect effect 0.198 (95 % CI 0.095–0.322) with significant dual mediation (b = 0.026; 95 % CI 0.004–0.066)
Atalla et al., 2024; Egypt<br><br>(Atalla et al., 2024)	Cross-sectional descriptive correlational study at Alexandria Main University Hospital, Egypt	415 registered nurses (347 female [83.6 %], 68 male [16.4 %])	Socio-demographic form<br>Perception of AI use scale (14 items; 5-point Likert)<br>General Attitudes Towards AI Scale (GAAIS; 20 items; 5-point Likert)<br>Ethical Awareness of AI scale (12	Descriptive statistics; independent t-tests and one-way ANOVA; Pearson correlations<br>Linear regression with interaction terms (SPSS v23): attitude positively predicted innovation (B = 1.796, p = 0.001) and ethical awareness did likewise (B = 2.567, p = 0.013); ethical awareness moderated the attitude–innovation link (interaction

			items; 5-point Likert)<br>Employee Innovative Behavior Scale (23 items; 5-point Likert)	B = 0.038, p = 0.002) but not the perception–innovation link (interaction B = 0.005, p = 0.767)
Kotp et al., 2025; Egypt (Kotp et al., 2025)	Descriptive cross-sectional study among nurse leaders (supervisors, CNOs, managers, educators, quality specialists) across nine private hospitals in Cairo, selected via multistage random and convenience sampling	187 nurse leaders	Nurse Leaders' Readiness for AI Integration Survey (20 items; 4-point Likert; three factors: Leadership Initiatives, Staff Engagement, Technical Readiness)<br>Perceived Benefits of AI-Driven Predictive Analytics Survey (10 items; 5-point Likert; two factors: Patient Care & Outcomes, Decision-Making Support)	EFA: three-factor readiness model (loadings 0.760–0.842) and two-factor benefits model (loadings 0.756–0.836)<br>CFA: readiness (χ^2/df=2.945; CFI=0.950; GFI=0.920; AGFI=0.892; RMSEA=0.069; RMR=0.054); benefits (χ^2/df=2.732; CFI=0.953; GFI=0.928; AGFI=0.887; RMSEA=0.067; RMR=0.050)<br>Reliability: Cronbach's α readiness=0.90 (test–retest r=0.854); benefits=0.89 (test–retest r=0.834)<br>Findings: 29.2% low, 34.0% moderate, 36.8% high readiness (mean low 65.05 ± 11.08); overall benefits mean 39.66 ± 4.32; readiness positively correlated with perceived benefits; age, education, and role significantly predicted readiness (R^2=0.375) and benefits (R^2=0.400)
Alruwaili et al., 2025; Saudi Arabia<br><br>(A. N. Alruwaili et al., 2025)	Interpretive phenomenological qualitative study across four high-risk Neonatal Intensive Care Units in the Eastern Region of Saudi Arabi	33 neonatal nurses (29 female [87.9 %], 4 male [12.1 %]; roles: 20 staff nurses, 8 charge nurses, 5 unit coordinators)	Semi-structured individual interview guide<br>Focus-group discussion guide<br>Demographic questionnaire<br>Field-notes template	Thematic analysis (Braun & Clarke six-phase) using NVivo, with inductive–deductive coding, investigator triangulation, member-checking, reflexive journaling, and peer debriefing. Five overarching themes emerged: (1) AI as a complementary "second opinion" preserving autonomy, (2) enhanced critical thinking and proactive risk assessment, (3) shifting nurse roles toward consultative strategists, (4) dependence on robust infrastructure, training, and clear protocols, (5) cultural and systemic factors shaping adoption, alongside pragmatic workarounds and recommendations for context-aware, EHR-integrated, user-friendly AI tools
Tuncer & Tuncer, 2024; Turkey (Tuncer & Tuncer, 2024)	Descriptive cross-sectional online survey of professional nurses who follow a nursing-focused Instagram account, using convenience sampling via Google Forms	288 registered nurses (260 female [90.3 %], 28 male [9.7 %])	Information Identification Questionnaire for ChatGPT and AI Programs (8 items on awareness, professional development, integration in care plans)<br>General Attitudes to Artificial Intelligence Scale (GAAIS; 20-item Turkish version; Positive/Negative subscales)	Descriptive statistics (frequencies, percentages, means ± SD); Pearson correlations; independent samples t-tests; one-way ANOVA with Bonferroni post-hoc; Kruskal–Wallis for non-parametric comparisons. Found that higher education level, AI knowledge and use were significantly associated with more positive AI attitudes; demographic and experience variables showed significant group differences in GAAIS scores
Sabra et al., 2023; Egypt	Descriptive correlational cross-sectional study at Qena	200 registered nurses (128	Personal data sheet (6 items: age, gender, social status, qualifications, years of	Descriptive statistics (frequencies, percentages; means ± SD); chi-square tests showed perception varied by gender and social status ($p<0.01$), and attitude varied by age ($p=0.047$),

(Sabra et al., 2023)	University Hospitals (medical, surgical, ICUs, and operation units)	female [64.0 %], 72 male [36.0 %])	experience, unit)<br>Perception of AI scale (14 items; 3 subscales: knowledge [4 items], advantages [5], problems [5]; 5-point Likert) by Abdullah (2020)<br>Attitude towards AI scale (14 items; 5-point Likert; negative vs positive scoring) by Sindermann et al. (2021)	qualifications ($p=0.005$), years of experience ($p=0.010$), and social status ($p<0.01$)
Alenazi & Alhalal, 2025; Saudi Arabia<br><br>(Alenazi & Alhalal, 2025)	Cross-sectional, multicentre survey at three public and private universities in Riyadh, Saudi Arabia	500 undergraduate nursing students (376 female [75.2 %], 124 male [24.8 %])	Arabic-adapted UTAUT2 questionnaire (30 items; subscales: performance expectancy 4; effort expectancy 4; social influence 3; facilitating conditions 4; hedonic motivation 3; price value 3; habit 3; behavioral intention 3; use behavior 3; all 5-point Likert)	Structural equation modeling with CFA and SEM: performance expectancy ($\beta = 0.235$, $p = 0.004$), facilitating conditions ($\beta = 0.233$, $p = 0.026$), hedonic motivation ($\beta = 0.371$, $p < 0.001$) and habit ($\beta = 0.458$, $p < 0.001$) predicted behavioral intention; behavioral intention ($\beta = 0.702$, $p < 0.001$), facilitating conditions ($\beta = 0.271$, $p = 0.006$) and habit ($\beta = 0.316$, $p < 0.001$) predicted actual use; intention mediated effects of performance expectancy, hedonic motivation and habit on use; gender moderated the effects of intention on use ($\Delta\beta = 0.613$, $p = 0.005$) and of facilitating conditions on use ($\Delta\beta = – 0.440$, $p = 0.023$)
Şimşek et al., 2025; Turkey<br><br>(Şimşek et al., 2025)	Descriptive–correlational study at Koç University (Istanbul) and Bilecik Şeyh Edebali University (Bilecik), Turkey	441 undergraduate nursing students (314 female [71.2 %], 127 male [28.8 %])	Nursing Students Competency Scale (CINS; 43 items, 6 subscales; 7-point Likert)<br>General Attitudes to Artificial Intelligence Scale (GAAIS; 20 items; 5-point Likert)<br>Generative Artificial Intelligence Acceptance Scale (GAIAS; 20 items; 5-point Likert)	Descriptive statistics and linear regression showed that facilitating conditions, social influence, and negative attitudes toward AI significantly predicted clinical competence; positive attitudes and acceptance accounted for 8.6 % of variance in competence ($R^2=0.086$, $p<0.01$)
Mariano et al., 2025; Saudi Arabia<br><br>(Mariano et al., 2025)	Cross-sectional descriptive correlational online survey via Google Forms, distributed by convenience and snowball sampling among staff nurses, nursing faculty, and students across four	349 staff nurses sparticipants (315 female [90.3 %], 34 male [9.7 %])	Sociodemographic form<br>AI knowledge test (10 MCQs; scored 0–1)<br>Attitudes toward AI scale (12 items; 5-point Likert)<br>Practice/experience in AI scale (12 items; 5-point Likert	Descriptive statistics (frequencies, means, SDs); Spearman's correlations showing significant positive associations between knowledge and attitudes ($r = 0.451$, $p < 0.01$), knowledge and practice ($r = 0.404$, $p < 0.01$), and attitudes and practice ($r = 0.439$, $p < 0.01$)

	government universities, two private medical colleges, and six public hospitals/clinics in Saudi Arabia			
Oweidat et al., 2025; Jordan (Oweidat et al., 2025)	Descriptive correlational cross-sectional survey at four governmental hospitals in Jordan (Princess Basma Teaching Hospital; Princess Raya Governmental Hospital; Ramtha Governmental Hospital; Yarmouk Governmental Hospital)	116 registered nurses (39 female [33.6 %], 77 male [66.4 %])	Sociodemographic form<br>AI engagement questionnaire: 40 items across four subscales (knowledge, attitudes, practices, barriers; 5-point Likert)<br>Intent to Stay scale: 10 items (5-point Likert)	Descriptive statistics (means, SDs, frequencies, percentages); Pearson correlations; multivariate linear regression ($R = 0.75$, $R^2 = 0.56$) identified attitudes ($\beta = 0.34$, $P < 0.001$), practices ($\beta = 0.29$, $P < 0.001$), and knowledge ($\beta = 0.22$, $P < 0.01$) as positive predictors of intent to stay, while barriers negatively predicted intent to stay ($\beta = –0.14$, $P < 0.05$)
Ünal & Avcı, 2024; Turkey<br><br>(Ünal & Avcı, 2024)	Cross-sectional descriptive survey via Google Forms of neonatal intensive care nurses in Ankara, Turkey	107 neonatal nurses (33 female [30.8 %], 74 male [69.2 %])	Sociodemographic form<br>Artificial Intelligence Anxiety Scale (AIAS; 16 items, four subscales: learning, job change, sociotechnical blindness, AI configuration; 5-point Likert; α=0.986)<br>Medical Artificial Intelligence Readiness Scale (MAIRS; 22 items, four sub-dimensions: cognitive, skill, foresight, ethical; 5-point Likert; α=0.984)	Descriptive statistics; scale reliabilities via Cronbach's α; Spearman's correlation between AIAS and MAIRS ($r = –0.549$, $p < 0.01$); Mann–Whitney U tests showing AIAS and MAIRS score differences by demographics and AI familiarity (all $p < 0.05$). Concluded that higher AI anxiety is linked to lower readiness, while higher education and AI knowledge predict greater readiness and reduced anxiety
Kwak, Ahn et al., 2022; Republic of Korea<br><br>(Kwak, Ahn, et al., 2022)	Cross-sectional survey at a nursing college in Gyeonggi-do, South Korea	189 nursing students (162 female [85.7 %], 27 male [14.3 %])	Test for Artificial Intelligence Ethics Awareness (TAIEA; 24 items; 5-point Likert)<br>General Attitudes Towards AI Scale (GAAIS; 20 items; 5-point Likert)<br>TAM-based scales: anxiety (4 items), self-efficacy (4 items), behavioral intention (3 items); all 5-point Likert	Descriptive statistics; t-tests; Pearson correlations; hierarchical multiple regression ($R^2 = 0.44$) showed positive attitude ($\beta = 0.49$, $p < 0.001$) and self-efficacy ($\beta = 0.22$, $p = 0.002$) significantly predict behavioral intention

Tsiara et al., 2025; Greece<br><br>(Tsiara et al., 2025)	Cross-sectional survey of undergraduate nursing students at the Department of Nursing, Faculty of Health Sciences, University of Thessaly, Larissa, Greece	159 nursing students (46 male [28.9 %], 112 female [70.4 %], 1 other [0.6 %])	General Attitudes Toward AI Scale (GAAIS; 20 items, two subscales: positive/negative attitudes; 5-point Likert)<br>Ten-Item Personality Inventory (TIPI; Big Five traits; 7-point Likert)	Descriptive statistics and reliability (Cronbach's α: GAAIS 0.790; negative subscale 0.670; TIPI subscales α 0.016–0.571)<br>Confirmatory factor analysis of GAAIS: χ^2/df 1.777; CFI 0.800; GFI 0.984; SRMR 0.083; RMSEA 0.070, supporting two-factor model<br>Pearson correlations: positive vs negative attitudes $r = -0.233$ ($p = 0.006$); Extraversion vs negative attitudes $r = -0.181$ ($p = 0.030$); Openness vs positive attitudes $r = 0.166$ ($p = 0.043$); maternal education inversely associated with negative attitudes ($F = 4.771$; $p = 0.010$)
Jalal et al., 2025; Saudi Arabia<br><br>(Jalal et al., 2025)	Cross-sectional survey at three major healthcare facilities in Al-Ahsa among registered nurses in ICUs, medical, surgical, emergency and outpatient units	246 registered nurses (188 female [76.4 %], 58 male [23.6 %])	guided by SPSS-based inferential framework<br><br>Structured questionnaire (validated by expert panel; pilot tested; Cronbach's α = 0.916) consisting of:<br>– Demographics (age, gender, education, role, experience, nationality, AI-IPC training)<br>– Knowledge of AI in IPC (20 MCQs; scored 0–20; categorized poor/average/good)<br>– Perceptions & attitudes (5 Likert items; 1–5; categorized negative/neutral/positive)<br>– Perceived barriers (closed-ended checklist: training, technical, ethical, resource, support)	Descriptive statistics (means, SDs, frequencies)<br>Chi-square tests for associations between knowledge level and demographics (age, education, role, AI training significant; no gender or nationality effect)<br>SPSS v21 used; $p < 0.05$ threshold<br>Findings highlight moderate overall knowledge but notable gaps in subdomains (surface disinfection, hand hygiene), generally positive yet cautious attitudes, and multilevel barriers emphasizing need for structured training, resource allocation, and ethical safeguards to optimize AI-driven IPC integration.
Chen et al., 2025 (China)<br><br>(Chen et al., 2025)	Descriptive qualitative study; semi-structured interviews conducted at three medical universities and three affiliated hospitals in Shanghai, Suzhou, and Chengdu	12 nursing professionals (educators and clinical practitioners)	Semi-structured interview guide	Qualitative content analysis yielded three overarching themes with 11 sub-themes:<br>1. Potential of AI-driven nursing: decision-support tools; documentation assistance; robots for high-risk or physically demanding tasks; embodied intelligence for care activities.<br>2. Multi-dimensional response: prioritizing education/research; exploring application scenarios; fostering deep interdisciplinary collaboration.<br>3. Obstacles: human–technology–machine interaction

				challenges; need for sustained funding; gaps in AI maturity; risk, safety, and fault-tolerance concerns

Legend:
This table summarizes the key features of each study included in the review, organized by: (1) study identification (author, year, country); (2) design and setting; (3) sample characteristics (N); (4) measures or instruments employed; and (5) analytical approaches and principal findings. Abbreviations used throughout are defined as follows:

N: sample size

GAAIS: General Attitudes towards Artificial Intelligence Scale

MAIRS-MS: Medical Artificial Intelligence Readiness Scale–Medical Students

AILS: Artificial Intelligence Literacy Scale

AIAS: Artificial Intelligence Anxiety Scale

UTAUT: Unified Theory of Acceptance and Use of Technology

TAM: Technology Acceptance Model

TIPI: Ten-Item Personality Inventory

CTSE: Career and Talent Development Self-Efficacy Scale

ANOVA: analysis of variance

t-test: Student’s t-test

EFA: exploratory factor analysis

CFA: confirmatory factor analysis

SEM: structural equation modeling (including path analysis)

PROCESS macro: Hayes’ PROCESS macro for mediation or moderation analysis

α: Cronbach’s alpha (internal consistency)

β: standardized regression coefficient

χ^2/df: chi-square statistic divided by degrees of freedom

CFI: comparative fit index

RMSEA: root mean square error of approximation

References

Abou Hashish, E. A., & Alnajjar, H. (2024). Digital proficiency: assessing knowledge, attitudes, and skills in digital transformation, health literacy, and artificial intelligence among university nursing students. *BMC Medical Education*, *24*(1), 1–11. https://doi.org/10.1186/S12909-024-05482-3/FIGURES/1

Ahmed, E., Ahmed, A., Yahia, M., Sayed, O., Farghaly, A., & Mohamed, A. (2024). Artificial Intelligence and the Future of Health Care: Is it threatening The Existence of Nursing? Nurses' Perception and Attitude. *Egyptian Journal of Health Care*, *15*(2), 1101–1114. https://doi.org/10.21608/EJHC.2024.363907

Akca Sumengen, A., Ozcevik Subasi, D., & Cakir, G. N. (2025). Nursing students' attitudes and literacy toward artificial intelligence: a cross-sectional study. *Teaching and Learning in Nursing*, *20*(1), e250–e257. https://doi.org/10.1016/J.TELN.2024.10.022

Al Omari, O., Alshammari, M., Al Jabri, W., Al Yahyaei, A., Aljohani, K. A., Sanad, H. M., Al-Jubouri, M. B., Bashayreh, I., Fawaz, M., ALBashtawy, M., Alkhawaldeh, A., Qaddumi, J., Shalaby, S. A., Abdallah, H. M., AbuSharour, L., Al Qadire, M., & Aljezawi, M. (2024). Demographic factors, knowledge, attitude and perception and their association with nursing students' intention to use artificial intelligence (AI): a multicentre survey across 10 Arab countries. *BMC Medical Education*, *24*(1), 1–11. https://doi.org/10.1186/S12909-024-06452-5/TABLES/6

Alenazi, L., & Alhalal, E. (2025). Factors affecting Artificial Intelligence usage intention among nursing students: Unified theory of acceptance and use of technology. *Nurse Education Today*, *152*, 106780. https://doi.org/10.1016/J.NEDT.2025.106780

Almagharbeh, W. T., Alfanash, H. A. K., Alnawafleh, K. A., Alasmari, A. A., Alsaraireh, F. A., Dreidi, M. M., & Nashwan, A. J. (2025). Application of artificial intelligence in nursing practice: a qualitative study of Jordanian nurses' perspectives. *BMC Nursing*, *24*(1), 1–14. https://doi.org/10.1186/S12912-024-02658-6/TABLES/8

Alruwaili, A. N., Alshammari, A. M., Alhaiti, A., Elsharkawy, N. B., Ali, S. I., & Elsayed Ramadan, O. M. (2025). Neonatal nurses' experiences with generative AI in clinical decision-making: a qualitative exploration in high-risk nicus. *BMC Nursing*, *24*(1), 1–20. https://doi.org/10.1186/S12912-025-03044-6/TABLES/2

Alruwaili, M. M., Abuadas, F. H., Alsadi, M., Alruwaili, A. N., Elsayed Ramadan, O. M., Shaban, M., Al Thobaity, A., Alkahtani, S. M., & El Arab, R. A. (2024). Exploring nurses' awareness and attitudes toward artificial intelligence: Implications for nursing practice. *Digital Health*, *10*. https://doi.org/10.1177/20552076241271803/SUPPL_FILE/SJ-DOCX-1-DHJ-10.1177_20552076241271803.DOCX

Atalla, A. D. G., El-Ashry, A. M., & Mohamed Sobhi Mohamed, S. (2024). The moderating role of ethical awareness in the relationship between nurses' artificial intelligence perceptions, attitudes, and innovative work behavior: a cross-sectional study. *BMC Nursing*, *23*(1), 1–11. https://doi.org/10.1186/S12912-024-02143-0/FIGURES/1

Chen, Y., Wu, F., Zhang, W., Xing, W., Zhu, Z., Huang, Q., & Yuan, C. (2025). Perspectives on AI-Driven Nursing Science Among Nursing Professionals from China: A Qualitative Study. *Nursing Reports 2025, Vol. 15, Page 218*, *15*(6), 218. https://doi.org/10.3390/NURSREP15060218

Cho, K. A., & Seo, Y. H. (2024). Dual mediating effects of anxiety to use and acceptance attitude of artificial intelligence technology on the relationship between nursing students' perception of and intention to use them: a descriptive study. *BMC Nursing*, *23*(1), 1–8. https://doi.org/10.1186/S12912-024-01887-Z/FIGURES/1

Demir-Kaymak, Z., Turan, Z., Unlu-Bidik, N., & Unkazan, S. (2024). Effects of midwifery and nursing students' readiness about medical Artificial intelligence on Artificial intelligence anxiety. *Nurse Education in Practice*, *78*. https://doi.org/10.1016/j.nepr.2024.103994

El-Sayed, B. K. M., El-Sayed, A. A. I., Alsenany, S. A., & Asal, M. G. R. (2025). The role of artificial intelligence literacy and innovation mindset in shaping nursing students' career and talent self-efficacy. *Nurse Education in Practice*, *82*, 104208. https://doi.org/10.1016/J.NEPR.2024.104208

Hamad, N. I., El-Ashry, A. M., Ibrahim, I. M., & Hassan, E. A. (2025). Embracing the future: an insight into nursing students' attitude and perception towards the usability of artificial intelligence in healthcare. *Teaching and Learning in Nursing*, *20*(2), e500–e508. https://doi.org/10.1016/J.TELN.2024.12.017

Hussein Mohamed, S., Abed El-Rahman Mohamed, M., Farouk Mahmoud, S., & HessienYousef Heggy, E. (2023). The Effect of Educational Program on Nurses' Knowledge and Attitude Regarding Artificial Intelligence. *Egyptian Journal of Health Care*, *14*(2), 1110–1128. https://doi.org/10.21608/EJHC.2023.312617

Jalal, S. M., Jalal, S. H., Alasmakh, K. E., Alnasser, Z. H., Alhamdan, W. Y., & Alabdullatif, A. A. (2025). Nurses' Perception of Artificial Intelligence-Driven Monitoring Systems for Enhancing Compliance With Infection Prevention and Control Measures in Al-Ahsa, Saudi Arabia. *Cureus*, *17*(4). https://doi.org/10.7759/CUREUS.82943

Kahraman, H., Akutay, S., Yüceler Kaçmaz, H., & Taşci, S. (2025). Artificial Intelligence Literacy Levels of Perioperative Nurses: The Case of Türkiye. *Nursing & Health Sciences*, *27*(1), e70059. https://doi.org/10.1111/NHS.70059;PAGEGROUP:STRING:PUBLICATION

Kotp, M. H., Ismail, H. A., Basyouny, H. A. A., Aly, M. A., Hendy, A., Nashwan, A. J., Hendy, A., & Abd Elmoaty, A. E. E. (2025). Empowering nurse leaders: readiness for AI integration and the perceived benefits of predictive analytics. *BMC Nursing*, *24*(1), 1–13. https://doi.org/10.1186/S12912-024-02653-X/TABLES/9

Kwak, Y., Ahn, J. W., & Seo, Y. H. (2022). Influence of AI ethics awareness, attitude, anxiety, and self-efficacy on nursing students' behavioral intentions. *BMC Nursing*, *21*(1), 1–8. https://doi.org/10.1186/S12912-022-01048-0/TABLES/4

Kwak, Y., Seo, Y. H., & Ahn, J. W. (2022). Nursing students' intent to use AI-based healthcare technology: Path analysis using the unified theory of acceptance and use of technology. *Nurse Education Today*, *119*. https://doi.org/10.1016/j.nedt.2022.105541

Labrague, L. J., Aguilar-Rosales, R., Yboa, B. C., Sabio, J. B., & de los Santos, J. A. (2023). Student nurses' attitudes, perceived utilization, and intention to adopt artificial intelligence (AI) technology in nursing practice: A cross-sectional study. *Nurse Education in Practice*, *73*. https://doi.org/10.1016/J.NEPR.2023.103815,

Lukić, A., Kudelić, N., Antičević, V., Lazić-Mosler, E., Glunčić, V., Hren, D., & Lukić, I. K. (2023). First-year nursing students' attitudes towards artificial intelligence: Cross-sectional multi-center study. *Nurse Education in Practice*, *71*, 103735. https://doi.org/10.1016/J.NEPR.2023.103735

Mariano, M. E. M., Shahin, M. A. H., Ancheta, S. J., Kunjan, M. V., Al Dossary, N. M., Al Ojaimi, S. F., Al Qudah, S. A., & Al Harbi, H. F. (2025). Exploring artificial intelligence knowledge, attitudes, and practices among nurses, faculty, and students in Saudi Arabia: A cross-sectional analysis. *Social Sciences & Humanities Open*, *11*, 101384. https://doi.org/10.1016/J.SSAHO.2025.101384

Oweidat, I. A., Alkhatib, M., ALBashtawy, M., Al Omar, S., Al-Rjoub, S., Alsaqer, K., Al-Mugheed, K., & Abdelaliem, S. M. F. (2025). Knowledge, attitudes, practices, and barriers of artificial intelligence as predictors of intent to stay among nurses: A cross-sectional study. *Digital Health*, *11*. https://doi.org/10.1177/20552076251336106/SUPPL_FILE/SJ-DOCX-1-DHJ-10.1177_20552076251336106.DOCX

Ramadan, O. M. E., Alruwaili, M. M., Alruwaili, A. N., Elsehrawy, M. G., & Alanazi, S. (2024). Facilitators and barriers to AI adoption in nursing practice: a qualitative study of registered nurses' perspectives. *BMC Nursing*, *23*(1), 1–16. https://doi.org/10.1186/S12912-024-02571-Y/FIGURES/3

Rony, M. K. K., Kayesh, I., Bala, S. Das, Akter, F., & Parvin, M. R. (2024). Artificial intelligence in future nursing care: Exploring perspectives of nursing professionals - A descriptive qualitative study. *Heliyon*, *10*(4), e25718. https://doi.org/10.1016/J.HELIYON.2024.E25718

Rony, M. K. K., Numan, S. M., Akter, K., Tushar, H., Debnath, M., Johra, F. tuj, Akter, F., Mondal, S., Das, M., Uddin, M. J., Begum, J., & Parvin, M. R. (2024). Nurses' perspectives on privacy and ethical concerns regarding artificial intelligence adoption in healthcare. *Heliyon*, *10*(17), e36702. https://doi.org/10.1016/J.HELIYON.2024.E36702

Rony, M. K. K., Numan, S. M., Johra, F. tuj, Akter, K., Akter, F., Debnath, M., Mondal, S., Wahiduzzaman, M., Das, M., Ullah, M., Rahman, M. H., Das Bala, S., & Parvin, M. R. (2024). Perceptions and attitudes of nurse practitioners toward artificial intelligence adoption in health care. *Health Science Reports*, *7*(8). https://doi.org/10.1002/HSR2.70006,

Sabra, H. E., Khalaf, H., Elaal, A., Sobhy, K. M., & Bakr, M. (2023). Utilization of Artificial Intelligence in Health Care: Nurses' Perspectives and Attitudes. *Menoufia Nursing Journal*, *8*(1), 253–268. https://doi.org/10.21608/MENJ.2023.297411

Salama, N., Bsharat, R., Alwawi, A., & Khlaif, Z. N. (2025). Knowledge, attitudes, and practices toward AI technology (ChatGPT) among nursing students at Palestinian universities. *BMC Nursing*, *24*(1), 1–17. https://doi.org/10.1186/S12912-025-02913-4/TABLES/13

Sarman, A., & Tuncay, S. (2025). Attitudes and anxiety levels of nursing students toward artificial intelligence. *Teaching and Learning in Nursing*, *20*(2), e431–e438. https://doi.org/10.1016/J.TELN.2024.12.006

Şimşek, E., Kudubeş, A. A., & Semerci Şahin, R. (2025). The predictive effect of nursing students' attitudes and acceptance towards artificial intelligence on their clinical competencies. *Teaching and Learning in Nursing*, *20*(3), e806–e814. https://doi.org/10.1016/J.TELN.2025.02.036

Summers, A., Haddad, M. El, Prichard, R., Clarke, K. A., Lee, J., & Oprescu, F. (2024). Navigating challenges and opportunities: Nursing student's views on generative AI in higher education. *Nurse Education in Practice*, *79*, 104062. https://doi.org/10.1016/J.NEPR.2024.104062

Tsiara, A., Bakalis, V. I., Toska, A., Zyga, S., Stathoulis, J. D., Albani, E. N., Saridi, M., Togas, C., Agraniotis, M., & Fradelos, E. C. (2025). The Role of Personality Traits in Nursing Students' Attitudes Toward Artificial Intelligence. *Cureus*, *17*(2). https://doi.org/10.7759/CUREUS.78847

Tuncer, G. Z., & Tuncer, M. (2024). Investigation of nurses' general attitudes toward artificial intelligence and their perceptions of ChatGPT usage and influencing factors. *DIGITAL HEALTH*, *10*. https://doi.org/10.1177/20552076241277025

Ünal, A. S., & Avcı, A. (2024). Evaluation of neonatal nurses' anxiety and readiness levels towards the use of artificial intelligence. *Journal of Pediatric Nursing*, *79*, e16–e23. https://doi.org/10.1016/J.PEDN.2024.09.012

Yalcinkaya, T., Ergin, E., & Yucel, S. C. (2024). Exploring Nursing Students' Attitudes and Readiness for Artificial Intelligence: A Cross-Sectional Study. *Teaching and Learning in Nursing*, *19*(4), e722–e728. https://doi.org/10.1016/J.TELN.2024.07.008
