# Supplementary material for: Artificial intelligence in nursing: a systematic review of attitudes, literacy, readiness, and adoption intentions among nursing students and practicing nurses
Source: Front Digit Health. 2025 Sep 25;7:1666005. doi: 10.3389/fdgth.2025.1666005 (PMC12507812; doi:10.3389/fdgth.2025.1666005)
Supplement: Supplementary file 3 [file Datasheet3.pdf]

Supplementary Material 3. Table 1. Quality Assessment (JBI Critical Appraisal – Analytical Cross-Sectional Studies)

Study	1. Inclusion criteria defined?	2. Subjects & setting described?	3. Exposure measured validly & reliably?	4. Standard criteria for outcome measurement?	5. Confounders identified?	6. Strategies to deal with confounding?	7. Outcomes measured validly & reliably?	8. Appropriate statistical analysis?	Total Score	Max Score	QA Rating
Lukić et al. 2023	Yes	Yes	Yes	Yes	Yes	Yes	Yes	Yes	8	8	High
Yalcınkaya et al. 2024	Yes	Yes	Yes	Yes	Yes	Yes	Yes	Yes	8	8	High
Kwak, Seo et al. 2022	Yes	Yes	Yes	Yes	Yes	Yes	Yes	Yes	8	8	High
Labrague et al. 2023	Yes	Yes	Yes	Yes	Yes	Yes	Yes	Yes	8	8	High
Demir-Kaymak et al. 2024	Yes	Yes	Yes	Yes	Yes	Yes	Yes	Yes	8	8	High
El-Sayed et al. (2025)	Yes	Yes	Yes	Yes	Yes	Yes	Yes	Yes	8	8	High
Akca Sumengen et al. (2025)	Yes	Yes	Yes	Yes	Yes	No	Yes	Yes	7	8	High
Sarman & Tuncay (2025)	Yes	Yes	Yes	Yes	Yes	No	Yes	Yes	7	8	High
Hamad et al. (2025)	Yes	Yes	Yes	Yes	Yes	Yes	Yes	Yes	8	8	High
Alruwaili et al. (2024)	Yes	Yes	Yes	Yes	Yes	No	Yes	Yes	7	8	High
Abou Hashish & Alnajjar (2024)	Yes	Yes	Yes	Yes	Yes	No	Yes	Yes	7	8	High
Ahmed et al. (2024)	Yes	Yes	Yes	Yes	Yes	No	Yes	Yes	7	8	High

Salama et al. (2025)	Yes	Yes	Yes	Yes	Yes	No	Yes	Yes	7	8	High
Kahraman H. et al. (2025)	Yes	Yes	Yes	Yes	Yes	No	Yes	Yes	7	8	High
Al Omari et al. (2024)	Yes	Yes	Yes	Yes	Yes	Yes	Yes	Yes	8	8	High
Cho & Seo (2024)	Yes	Yes	Yes	Yes	Yes	No	Yes	Yes	7	8	High
Atalla et al. (2024)	Yes	Yes	Yes	Yes	Yes	Yes	Yes	Yes	8	8	High
Kotp et al. (2025)	Yes	Yes	Yes	Yes	Yes	Yes	Yes	Yes	8	8	High
Tuncer & Tuncer (2024)	Yes	Yes	Yes	Yes	Yes	No	Yes	Yes	7	8	High
Sabra HE et al. (2023)	Yes	Yes	Yes	Yes	Yes	No	Yes	Yes	7	8	High
Alenazi & Alhalal (2025)	Yes	Yes	Yes	Yes	Yes	No	Yes	Yes	7	8	High
Şimşek et al. (2025)	Yes	Yes	Yes	Yes	No	No	Yes	Yes	6	8	Moderate
Mariano et al. (2025)	Yes	Yes	Yes	Yes	Yes	No	Yes	Yes	7	8	High
Oweidat et al. (2025)	Yes	Yes	Yes	Yes	Yes	Yes	Yes	Yes	8	8	High
Ünal & Avcı (2024)	Yes	Yes	Yes	Yes	Yes	No	Yes	Yes	7	8	High
Kwak, Ahn et al. (2022)	Yes	Yes	Yes	Yes	Yes	No	Yes	Yes	7	8	High
Tsiara et al. (2025)	Yes	Yes	Yes	Yes	Yes	No	Yes	Yes	7	8	High
Jalal et al. (2025)	Yes	Yes	Yes	Yes	Yes	No	Yes	Yes	7	8	High

Supplementary Material 3. Table 2. Quality Assessment (JBI Critical Appraisal Checklist for Qualitative Research)

Study (Author, Year)	1. Philosophical congruity	2. Method – question congruity	3. Method –data collection congruity	4. Method – analysis congruity	5. Researcher positioning	6. Reflexivity addressed	7. Participants' voices represented	8. Ethical approval	9. Conclusions flow from data	10. Themes adequately derived	Total Score	Max Score	QA Ratin
Summers et al. 2024	No	Yes	Yes	Yes	No	Unclear	Yes	Yes	Yes	Yes	7	10	Moderate
Rony, Kayesh, et al., 2024	No	Yes	Yes	Yes	No	Unclear	Yes	Yes	Yes	Yes	7	10	Moderate
Ramadan et al. (2024)	No	Yes	Yes	Yes	Yes	No	Yes	Yes	Yes	Yes	8	10	High
Almagharbeh et al. (2025)	No	Yes	Yes	Yes	No	No	Yes	Yes	Yes	Yes	7	10	Moderate
Rony, Numan, Johra, et al., 2024	Yes	Yes	Yes	Yes	Yes	No	Unclear	Yes	Yes	Yes	8	10	High (8–10)
Rony, Numan, Akter, et al., 2024	Yes	Yes	Yes	Yes	Yes	Yes	Yes	Yes	Yes	Yes	10	10	High (8–10)
Alruwaili et al. (2025)	Yes	Yes	Yes	Yes	No	Yes	Yes	Yes	Yes	Yes	9	10	High
Chen et al. (2025)	No	Yes	Yes	Yes	No	Yes	Yes	Yes	Yes	Yes	8	10	High (8–10)

Supplementary Material 3. Table 3. Quality Assessment (JBI Critical Appraisal Checklist for Quasi-Experimental Studies)

Study (Author, Year)	1. Clear cause – effect ?	2. Comparable participants ?	3. Similar care other than intervention ?	4. Control group?	5. Multiple pre/post measurements ?	6. Complete follow-up?	7. Same outcome measurement ?	8. Reliable measurement ?	9. Appropriate analysis?	Total Score	Max Score	QA Rating
Mohamed et al. 2023	Yes	Yes	Yes	No	Yes	Yes	Yes	Yes	Yes	8	9	High (7–9)
