# Supplementary material for: Artificial intelligence in nursing: a systematic review of attitudes, literacy, readiness, and adoption intentions among nursing students and practicing nurses
Source: Front Digit Health. 2025 Sep 25;7:1666005. doi: 10.3389/fdgth.2025.1666005 (PMC12507812; doi:10.3389/fdgth.2025.1666005)
Supplement: Supplementary file 4 [file Datasheet4.pdf]

Supplementary Material 4. Table 1. Risk-of-Bias Assessment (cross-sectional)

Study	Selection Bias	Performance Bias	Detection Bias	Attrition Bias	Reporting Bias	Confounding	Overall RoB
lukić et al. 2023	Moderate	Low	Low	Low	Low	Moderate	Moderate
Yalcınkaya et al. 2024	Low	Low	Low	Low	Low	Moderate	Moderate
Kwak, Seo et al. 2022	Low	Low	Low	Low	Low	Low	Low
Labrague et al. 2023	Moderate	Low	Low	Low	Low	Moderate	Moderate
Demir-Kaymak Z et al. 2024	Moderate	Low	Low	Low	Low	Low	Moderate
El-Sayed et al. (2025)	Low	Low	Low	Low	Low	Low	Low
Akca Sumengen et al. (2025)	Moderate	Low	Low	Moderate	Low	Moderate	Moderate
Sarman & Tuncay (2025)	Low	Low	Low	Low	Low	Moderate	Moderate
Hamad et al. (2025)	Moderate	Low	Low	Low	Low	Low	Moderate
Alruwaili et al. (2024)	Moderate	Low	Low	Moderate	Low	Moderate	Moderate
Abou Hashish & Alnajjar (2024)	Moderate	Low	Low	Low	Low	Moderate	Moderate
Ahmed et al. (2024)	Moderate (convenience sample)	Low	Low	Low	Low	Moderate	Moderate
Salama et al. (2025)	Moderate	Low	Low	Low	Low	Moderate	Moderate
Kahraman H. et al. (2025)	Moderate	Low	Low	Low	Low	Moderate	Moderate
Al Omari et al. (2024)	Moderate	Low	Low	Low	Low	Low	Moderate
Cho & Seo (2024)	Moderate (convenience sampling)	Low	Low	Low	Low	Moderate	Moderate
Atalla et al. (2024)	Moderate	Low	Low	Low	Low	Low	Moderate
Kotp et al. (2025)	Moderate	Low	Low	Low	Low	Low	Moderate
Tuncer & Tuncer (2024)	Moderate	Low	Low	Low	Low	Moderate	Moderate
Sabra et al. (2023)	Moderate	Low	Low	Low	Low	Moderate	Moderate
Alenazi & Alhalal (2025)	Moderate	Low	Low	Low	Low	Moderate	Moderate
Şimşek et al. (2025)	Moderate	Low	Low	Low	Low	Moderate	Moderate
Mariano et al. (2025)	Moderate	Low	Low	Low	Low	Moderate	Moderate
Oweidat et al. (2025)	Moderate	Low	Low	Moderate	Low	Low	Moderate
Ünal & Avcı (2024)	Moderate	Low	Low	Low	Low	Moderate	Moderate
Kwak, Ahn et al. (2022)	Moderate	Low	Low	Low	Low	Moderate	Moderate
Tsiara et al. (2025)	Moderate	Low	Low	Low	Low	Moderate	Moderate
Jalal et al. (2025)	Low	Low	Low	Low	Low	Moderate	Moderate

Supplementary Material 4.Table 2. Risk-of-Bias Assessment (qualitative studies)

Study (Author, Year)	Selection Bias	Investigator Bias	Data-Collection Bias	Analysis Bias	Reporting Bias	Overall RoB
Summers et al. 2024	Moderate (self-selection)	Moderate (no reflexivity)	Low (standard protocol)	Low (team consensus)	Low (full reporting)	Moderate
Rony, Kayesh, et al., 2024	Moderate (purposive)	Moderate (no reflexivity)	Low (uniform protocol)	Low (systematic coding)	Low (full transparency)	Moderate
Ramadan et al. (2024)	Moderate – purposive sampling across four sites	Low – reflexive journaling & peer debriefing	Low – standardized focus-group guide, validated and back-translated	Low – independent coding by two researchers with consensus checks	Low – full, transparent reporting of themes and exemplar quotes	Moderate
Almagharbeh et al. (2025)	Moderate (purposive)	Moderate (no reflexivity)	Low (standard guides)	Low (team consensus)	Low (full transparency)	Moderate
Rony, Numan, Johra, et al., 2024	Moderate (purposive)	Moderate (no reflexivity)	Low (uniform protocol)	Low (team consensus)	Low (transparent)	Moderate
Rony, Numan, Akter, et al., 2024	Moderate	Low	Low	Low	Low	Moderate
Alruwaili et al. (2025)	Moderate	Low	Low	Low	Low	Moderate
Chen et al. (2025)	Moderate	Low	Low	Low	Low	Moderate

Supplementary Material 4. Table 3. Risk-of-Bias Assessment (ROBINS-I)

Study (Author, Year)	RoB Tool	Confounding	Selection of participants	Classification of intervention	Deviations from intended	Missing data	Measurement of outcomes	Selection of reported results	Overall RoB
Mohamed et al.2023	ROBINS-I	Moderate	Low	Low	Low	Low	Low	Low	Moderate
